# Virtual Screening of Phytochemicals From Medicinal Plants as Promising PDE5 Inhibitors Against Erectile Dysfunction

**DOI:** 10.1002/fsn3.71478

**Published:** 2026-02-01

**Authors:** Farouk Boudou, Alaeddine Berkane, Amal Belakredar, Ahcene Keziz, Huda Alsaeedi, Brian A. Murray, Mikhael Bechelany, Ahmed Barhoum

**Affiliations:** ^1^ Department of Applied Molecular Genetics, Faculty of Natural and Life Sciences University of Science and Technology of Oran Oran Algeria; ^2^ Laboratory of Chemistry, Synthesis, Properties, and Applications (LCSPA), Department of Chemistry, Faculty of Sciences Dr. Moulay Tahar University of Saida Saida Algeria; ^3^ Department of Biotechnology, Faculty of Natural Sciences and Life University of Mostaganem Abdelhamid Ibn Badis Mostaganem Algeria; ^4^ Department of Physics, and Chemistry of Materials Laboratory University of M'sila M'sila Algeria; ^5^ Department of Chemistry College of Science, King Saud University Riyadh Saudi Arabia; ^6^ Chemical and BioPharmaceutical Sciences Technological University Dublin Dublin Ireland; ^7^ Institut Européen Des Membranes (IEM), UMR 5635, Univ Montpellier, ENSCM, CNRS Montpellier France; ^8^ NanoStruc Research Group, Chemistry Department, Faculty of Science Helwan University Cairo Egypt; ^9^ School of Chemical and BioPharmaceutical Sciences Technological University Dublin Dublin Ireland; ^10^ Nanolab Research Centre, Physical to Life Sciences Research Hub Technological University Dublin Dublin Ireland

**Keywords:** algerian medicinal plants, drug‐likeness, erectile dysfunction, molecular docking, molecular dynamics, PDE5 inhibition, QSAR, toxicity assessment

## Abstract

This study evaluates bioactive phytochemicals from Algerian medicinal plants as potential phosphodiesterase‐5 (PDE5) inhibitors for the treatment of erectile dysfunction (ED) using an integrated in silico approach. A total of 76 compounds from 48 plant species were screened for drug‐likeness using SwissADME. Overall, 72% of the compounds complied with Lipinski's Rule of Five, indicating favorable oral bioavailability, while toxicity prediction identified 29 non‐toxic candidates. Molecular docking was validated by redocking the co‐crystallized PDE5 ligand (RMSD = 0.264 Å). Ellagic acid (−9.4 kcal·mol^−1^), rosmarinic acid (−9.2 kcal·mol^−1^), salvinorin A (−9.2 kcal·mol^−1^), and catechin (−9.0 kcal·mol^−1^) exhibited the strongest binding affinities. Molecular dynamics simulations revealed stable hydrogen‐bond interactions for rosmarinic acid, while salvinorin A showed compact and low‐fluctuation behavior. MM‐GBSA analysis confirmed favorable binding free energies for salvinorin A (−26.7 kcal·mol^−1^) and rosmarinic acid (−23.6 kcal·mol^−1^). A QSAR model based on docking‐derived pKd values and molecular descriptors showed strong predictive performance using Random Forest regression (*R* ^2^_train_ = 0.91; *R* ^2^_CV_ = 0.87), identifying LogP, molecular weight, and TPSA as key determinants of PDE5 inhibition. Overall, this study highlights catechin and related phytochemicals as promising natural PDE5 inhibitors, supporting their further preclinical evaluation as safer and affordable ED therapies.

## Graphical abstract

Phytochemicals from Algerian medicinal plants were virtually screened as potential phosphodiesterase‐5 (PDE5) inhibitors for erectile dysfunction therapy. Drug‐likeness and toxicity analyses identified catechin, ellagic acid, and rosmarinic acid as safe, bioavailable candidates. Molecular docking and QSAR modeling revealed strong correlations between binding affinity, LogP, and molecular weight. Molecular dynamics simulations confirmed catechin's stable binding to PDE5, supporting its potential as a natural, safe, and affordable PDE5 inhibitor.

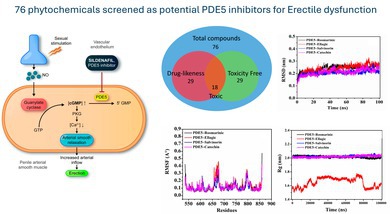

## 1 Introduction

Erectile dysfunction (ED) is a prevalent condition that significantly impacts male reproductive and psychological health, with incidence increasing with age. In severe cases, ED may contribute to infertility and reduced quality of life (Agarwal et al. 2015). Its etiology is multifactorial, including lifestyle factors (e.g., smoking, stress, obesity) and medical conditions such as diabetes and hypertension (Alahmar 2019; Shamloul and Ghanem 2013). Clinical manifestations range from ejaculatory disorders and reduced libido to the complete inability to maintain an erection, making ED a major public health concern. Currently, phosphodiesterase‐5 (PDE5) inhibitors such as sildenafil (Viagra), tadalafil, and vardenafil represent the first‐line therapy for ED. These drugs function by inhibiting PDE5‐mediated degradation of cyclic guanosine monophosphate (cGMP), thereby promoting penile vasodilation and erection (Guay et al. 2001). However, their clinical use is limited by side effects such as headache, back pain, and visual disturbances, resulting from off‐target interactions with other PDE isoforms (Foresta et al. 2008). Thus, there is growing demand for safer, long‐term alternatives, particularly from natural sources.

Herbal medicine has gained global attention as a safer alternative to synthetic drugs, attributed to its antioxidant, anti‐inflammatory, and phytoestrogenic properties. An estimated 60%–80% of the global population, particularly in developing countries, relies on plant‐based remedies (Thomford et al. 2015). Several studies report that plant extracts can significantly improve erectile function, in some cases by up to 96% (Jaradat and Zaid 2019). Additionally, phytotherapies often reduce adverse effects by 40%–60% and are up to five times more affordable compared to conventional PDE5 inhibitors (Dehelean et al. 2021). Nevertheless, drug discovery from natural sources faces challenges such as high failure rates, limited efficacy, and toxicity issues (Sang et al. 2018). Computational approaches, including molecular docking, QSAR modeling, and molecular dynamics (MD) simulations, have emerged as essential tools in modern drug discovery, enabling rapid and cost‐effective screening of phytochemicals for desirable pharmacokinetic and binding properties (Verma et al. 2021). This study aims to systematically screen bioactive compounds from Algerian medicinal plants for PDE5 inhibition. These plants, deeply rooted in traditional medicine, represent an untapped pharmacological resource. Specifically, we evaluated drug‐likeness, toxicity, molecular docking, QSAR modeling, and MD simulations to identify promising PDE5 inhibitors. By integrating computational screening with traditional knowledge, this study highlights the potential of Algerian phytochemicals to provide safer, natural alternatives to conventional PDE5 inhibitors (Rahman et al. 2024).

## 2 Experimental Section

### 2.1 Selection of Potential Ligands

A collection of 76 natural compounds from 48 medicinal plants, previously identified for their potential in treating human infertility, was selected for this study (Table 1). These compounds were sourced from previous studies conducted in Algeria (Adamu et al. 2012, 2014; Djerrou et al. 2022). The three‐dimensional (3D) structures of these compounds were retrieved from the PubChem database (Li et al. 2010), and their SMILES notation was generated to serve as input for the computational tools. The drug‐likeness and toxicity risk of these compounds were assessed to ensure the safety and viability of potential therapeutic candidates.

**TABLE 1:** Drug‐likeness prediction of selected herbal compounds (*n* = 76) using SwissADME.

Plant source	Ligand (formula)	Ref.	Molecular weight (MW) (g/mol)	Numberof H‐bond acceptors (HBA)	Numberof H‐bond donors (HBD)	Molar refractivity (MR)	MLOGP	WLOGP	Lipinski's Rule of Five	Ghose filter
*Allium sativum*	Alliin (C_6_H_11_NO_3_S)	Oyaluna et al. (2024)	177.22	4	2	43.24	−2.88	0.20	Yes; 0 violation	Yes
*Allium sativum*	Allicin (C_6_H_10_OS_2_)	Oyaluna et al. (2024)	162.27	1	0	45.88	1.18	2.62	Yes; 0 violation	No; 1 violation: < 20 atoms
*Allium sativum*	Ajoene (C_9_H_14_OS_3_)	Oyaluna et al. (2024)	234.40	1	0	67.41	2.10	3.87	Yes; 0 violation	Yes
*Allium sativum*	Vinyldithiin (C_6_H_6_S_2_)	Oyaluna et al. (2024)	142.24	1	0	42.60	1.86	2.97	Yes; 0 violation	No; 2 violations: MW < 160, < 20 atoms
*Arbutus unedo*	Arbutin (C_12_H_16_O_7_)	Bhalla et al. (2022)	272.25	7	5	62.61	−1.49	−1.43	Yes; 0 violation	No; 1 violation: WLOGP<‐0.4
*Calendula officinalis*	Calenduladiol (C_30_H_50_O_2_)	Vella et al. (2024)	442.72	2	2	136.30	6.00	7.00	Yes; 1 violation: MLOGP > 4.15	No; 3 violations: WLOGP > 5.6, MR > 130, > 70 atoms
*Calendula officinalis*	Officinoside A (C_19_H_32_O_8_)	Vella et al. (2024)	388.45	8	5	95.47	−0.85	−0.54	Yes; 0 violation	No; 1 violation: WLOGP<‐0.4
*Capsicum annuum*	Capsaicin (C_18_H_27_NO_3_)	Kuzma et al. (2014)	305.41	3	2	90.52	2.69	3.64	Yes; 0 violation	Yes
*Capsicum annuum*	Dihydrocapsaicin (C_18_H_29_NO_3_)	Kuzma et al. (2014)	307.43	3	2	90.99	2.78	3.86	Yes; 0 violation	Yes
*Artemisia herba‐alba*	Herbolide A (C_17_H_24_O_4_)	Mohammed et al. (2021)	292.37	4	0	81.23	2.83	3.17	Yes; 0 violation	Yes
*Artemisia herba‐alba*	Arteanoflavone (C_19_H_18_O_8_)	Mohammed et al. (2021)	374.34	8	2	97.93	−0.12	2.91	Yes; 0 violation	Yes
*Capsella bursa‐pastoris*	Diosmetin (C_16_H_12_O_6_)	Zhou et al. (2023)	300.26	6	3	80.48	0.22	2.59	Yes; 0 violation	Yes
*Lepidium sativum*	Lepidine B (C_20_H_18_N_4_O_2_)	Painuli et al. (2022)	346.38	4	3	98.18	1.33	3.81	Yes; 0 violation	Yes
*Lepidium sativum*	Semilepidinoside B (C_17_H_22_N_2_O_7_)	Painuli et al. (2022)	366.37	8	5	88.68	−1.58	−0.81	Yes; 0 violation	No; 1 violation: WLOGP<‐0.4
*Lepidium sativum*	Syringoylglucose (C_15_H_20_O_1_0)	Painuli et al. (2022)	360.31	10	6	81.69	−2.95	−2.40	Yes; 1 violation: NH or OH > 5	No; 1 violation: WLOGP < 0.4
*Ericacinerea*	Chrysanthemin (C_21_H21O_11_^+^)	Aires and Carvalho (2017)	449.38	11	8	108.29	−1.76	0.38	No; 2 violations: N or O > 10, NH or OH > 5	Yes
*Bryonia dioica*	Bryonioside A (C_36_H_60_O_9_)	Ukiya et al. (2002)	636.86	9	6	172.46	1.66	3.50	No; 2 violations: MW > 500, NH or OH > 5	No; 3 violations: MW > 480, MR > 130, > 70 #atoms
*Lavandula officinalis*	Rosmarinate (C_18_H_16_O_8_)	Mansour et al. (2021)	360.31	8	5	91.40	0.90	1.65	Yes; 0 violation	Yes
*Anthemis nobilis*	Apigenin (C_15_H_10_O_5_)	Mouissi et al. (2022)	270.24	5	3	73.99	0.52	2.58	Yes; 0 violation	Yes
*Daucus carota*	Carotamine (C_14_H_13_N_3_O)	Boadi et al. (2021)	271.27	3	4	76.42	0.06	1.63	Yes; 0 violation	Yes
*Cuminum cyminum*	Cuminoside A (C_21_H_30_O_8_)	Karik et al. (2021)	410.46	8	4	101.80	0.21	0.18	Yes; 0 violation	Yes
*Cuminum cyminum*	Cumene (C_9_H_12_)	Karik et al. (2021)	120.19	0	0	41.02	4.17	2.81	Yes; 1 violation: MLOGP > 4.15	No; 1 violation: MW < 160
*Melissa officinalis*	Melitric acid A (C_27_H_22_O_12_)	Zam et al. (2022)	538.46	12	7	136.10	0.77	2.75	No; 3 violations: MW > 500, N or O > 10, NH or OH > 5	No; 2 violations: MW > 480, MR > 130
*Foeniculum vulgare*	Avicularin (C_20_H_18_O_11_)	Rafieian et al. (2024)	434.35	11	7	104.19	−2.06	0.10	No; 2 violations: N or O > 10, NH or OH > 5	Yes
*Origanum vulgare*	Amburoside A (C_20_H_22_O_10_)	Puvača et al. (2022)	422.38	10	6	100.40	−0.83	−0.52	Yes; 1 violation: NH or OH > 5	No; 1 violation: WLOGP<‐0.4
*Zingiber officinalis*	Zingiberene (C_15_H_24_)	Yang et al. (2024)	204.35	0	0	70.68	4.53	4.89	Yes; 1 violation: MLOGP > 4.15	Yes
*Zingiber officinalis*	Gingerdiol (C_17_H_28_O_4_)	Yang et al. (2024)	296.40	4	3	85.51	2.23	3.03	Yes; 0 violation	Yes
*Zingiber officinalis*	(S)‐6‐gingerol (C_17_H_26_O_4_)	Yang et al. (2024)	294.39 g/mol	4	2	84.55	2.14	3.23	Yes; 0 violation	Yes
*Equisetum arvense*	Herbacitrin (C_21_H_20_O_12_)	Boeing et al. (2021)	464.38	12	8	110.16	−2.59	−0.54	No; 2 violations: N or O > 10, NH or OH > 5	No; 1 violation: WLOGP<‐0.4
*Lactuca virosa*	Scorzoside (C_21_H_30_O_8_)	Abdel Bar et al. (2023)	410.46	8	4	101.76	0.21	−0.11	Yes; 0 violation	Yes
*Rosmarinus officinalis*	Rosmarinic acid (C_18_H_16_O_8_)	Puvača et al. (2022)	360.31	8	5	91.40	0.90	1.65	Yes; 0 violation	Yes
*Rosmarinus officinalis*	Carnosic acid (C_20_H_28_O_4_)	Puvača et al. (2022)	332.43	4	3	95.43	3.25	4.32	Yes; 0 violation	Yes
*Rosmarinus officinalis*	Carnosol (C_20_H_26_O_4_)	Puvača et al. (2022)	330.42	4	2	92.83	3.25	3.96	Yes; 0 violation	Yes
*Marrubium vulgare*	Marrubin (C_20_H_28_O_4_)	Zahedifar and Najafian (2023)	332.43	4	1	91.40	2.76	3.72	Yes; 0 violation	Yes
*Marrubium vulgare*	Peregrinol (C_20_H_36_O_2_)	Zahedifar and Najafian (2023)	308.50	2	2	95.39	4.70	3.93	Yes; 0 violation	Yes
*Satureja hortensis*	Carvacrol (C_10_H_14_O)	Ejaz et al. (2023)	150.22	1	1	48.01	2.76	2.82	Yes; 0 violation	No; 1 violation: MW < 160
*Salvia officinalis*	Safficinolide (C_20_H_24_O_5_)	Ejaz et al. (2023)	344.40	5	1	94.14	2.25	3.51	Yes; 0 violation	Yes
*Salvia officinalis*	Columbaridione (C_20_H_24_O_5_)	Ejaz et al. (2023)	344.40	5	1	91.59	1.80	3.04	Yes; 0 violation	Yes
*Salvia officinalis*	Sageone (C_19_H_24_O_3_)	Ejaz et al. (2023)	300.39	3	2	89.49	2.98	4.31	Yes; 0 violation	Yes
*Myrthus communis*	Sideroxylin (C_18_H_16_O_5_)	Medda and Mulas (2021)	312.32	5	2	88.39	1.25	3.50	Yes; 0 violation	Yes
*Vinca minor*	Vincamine (C_21_H_26_N_2_O_3_)	Neculai et al. (2023)	354.44	4	1	103.74	2.62	2.14	Yes; 0 violation	Yes
*Vinca minor*	Vincaminoreine (C_22_H_30_N_2_O_2_)	Neculai et al. (2023)	354.49	3	0	109.62	3.26	3.49	Yes; 0 violation	Yes
*Nigella sativa*	Nigeglanine (C_12_H_14_N_2_O)	Manoharan et al. (2021)	202.25	1	0	61.19	1.69	1.91	Yes; 0 violation	Yes
*Nigella sativa*	Nigellidine (C_18_H_18_N_2_O_2_)	Manoharan et al. (2021)	294.35	2	1	88.65	2.39	3.28	Yes; 0 violation	Yes
*Urtica dioica*	Enterofuran (C_18_H_20_O_3_)	Đurović et al. (2024)	284.35	3	2	82.95	2.66	3.15	Yes; 0 violation	Yes
*Urtica dioica*	Neoolivil (C_20_H_24_O_7_)	Đurović et al. (2024)	376.40	7	4	98.25	0.37	1.50	Yes; 0 violation	Yes
*Petroselinum sativum*	Isopimpinellin (C_13_H_10_O_5_)	Tahraoui et al. (2007)	246.22	5	2	65.24	0.89	2.56	Yes; 0 violation	Yes
*Ajuga iva* L.	Clerodane (C_20_H_38_)	Djordjevic et al. (2018)	278.52	0	0	93.51	6.69	6.99	Yes; 1 violation: MLOGP > 4.15	No; 1 violation: WLOGP > 5.6
*Teucrium polium* L.	Luteolin (C_15_H_10_O_6_)	Djordjevic et al. (2018)	286.24	6	4	76.01	−0.03	2.28	Yes; 0 violation	Yes
*Teucrium polium* L.	Apigenin (C_15_H_10_O_5_)	Djordjevic et al. (2018)	270.24	5	3	73.99	0.52	2.58	Yes; 0 violation	Yes
*Artemisia herba alba Asso*	Carvone (C_10_H_14_O)	Tahraoui et al. (2007)	150.22	1	0	47.32	2.10	2.49	Yes; 0 violation	No; 1 violation: MW < 160
*Artemisia herba alba Asso*	Piperitone (C_10_H_16_O)	Tahraoui et al. (2007)	152.23	1	0	47.80	2.20	2.57	Yes; 0 violation	No; 1 violation: MW < 160
*Malva sylvestris* L.	2‐Methoxy‐4‐vinylphenol (C_9_H_10_O_2_)	Dushkin et al. (2014)	150.17	2	1	45.05	1.71	1.93	Yes; 0 violation	No; 1 violation: MW < 160
*Glycyrrhiza glabra* L.	Glycyrrhizin (C_42_H_62_O_16_)	Dushkin et al. (2014)	822.93	16	8	202.84	0.02	2.25	No; 3 violations: MW > 500, N or O > 10, NH or OH > 5	No; 3 violations: MW > 480, MR > 130, > 70 #atoms
*Punica granatum* L.	Punicalagin (C_48_H_28_O_3_0)	Dushkin et al. (2014)	1084.72	30	17	250.86	−3.29	1.96	No; 3 violations: MW > 500, N or O > 10, NH or OH > 5	No; 3 violations: MW > 480, MR > 130, > 70 #atoms
*Punica granatum* L.	Ellagic acid (C_14_H_6_O_8_)	Dushkin et al. (2014)	302.19	8	4	75.31	0.14	1.31	Yes; 0 violation	Yes
*Juniperus oxyedrus*	Alpha‐pinene (C_10_H_16_)	Zouaoui et al. (2024)	136.23	0	0	45.22	3.00	4.29	Yes; 1 violation: MLOGP > 4.15	No; 1 violation: MW < 160
*Salvia officinalis* L.	Carnosol (C_20_H_26_O_4_)	Tahraoui et al. (2007)	330.42	4	2	92.83	3.25	3.96	Yes; 0 violation	Yes
*Salvia officinalis* L.	Salvinorin A (C_23_H_28_O_8_)	Tahraoui et al. (2007)	432.46	8	0	107.39	1.56	2.68	Yes; 0 violation	Yes
*Salvia officinalis* L.	Rosmarinic acid (C_18_H_16_O_8_)	Tahraoui et al. (2007)	360.31	8	5	91.40	0.90	1.65	Yes; 0 violation	Yes
*Quercus ilex*	Catechin (C_15_H_14_O_6_)	Sánchez‐Gutiérrez et al. (2022)	290.27	6	5	74.33	1.22	0.24	Yes; 0 violation	Yes
*Quercus ilex*	Quercetin (C_15_H_10_O_7_)	Sánchez‐Gutiérrez et al. (2022)	302.24	7	5	78.03	−0.56	1.99	Yes; 0 violation	Yes
*Quercus ilex*	Gallic acid (C_7_H_6_O_5_)	Sánchez‐Gutiérrez et al. (2022)	170.12	5	4	39.47	−0.16	0.50	Yes; 0 violation	No; 2 violations: MR < 40, < 20 #atoms
*Quercus ilex*	Kaempferol (C_15_H_10_O_6_)	Sánchez‐Gutiérrez et al. (2022)	286.24	6	4	76.01	−0.03	2.28	Yes; 0 violation	Yes
*Artemisia campestris*	Limonene (C_10_H_16_)	Kemal et al. (2023)	136.23	0	0	47.12	3.27	3.31	Yes; 0 violation	No; 1 violation: MW < 160
*Artemisia campestris*	Beta‐pinene (C_10_H_16_)	Kemal et al. (2023)	136.23	0	0	45.22	4.29	3.00	Yes; 1 violation: MLOGP > 4.15	No; 1 violation: MW < 160
*Artemisia campestris*	Myrcene (C_10_H_17_Cl)	Kemal et al. (2023)	136.23	0	0	48.76	3.56	3.48	Yes; 0 violation	No; 1 violation: MW < 160
*Lawsonia alba* L	Naphthoquinone (C_10_H_6_O_2_)	Mustapha et al. (2024)	158.15	2	0	44.24	0.91	1.62	Yes; 0 violation	No; 2 violations: MW < 160, < 20 #atoms
*Pinus halepensis*	Alpha‐humulene (C_15_H_24_)	Fekih et al. (2014)	204.35	0	0	4.53	5.04		Yes; 1 violation: MLOGP > 4.15	Yes
*Pinus halepensis*	Phenyl 3‐methylbutanoate (C_11_H_14_O_2_)	Fekih et al. (2014)	178.23	2	0	52.36	2.84	2.64	Yes; 0 violation	Yes
*Pinus halepensis*	Sabinene (C_10_H_16_)	Fekih et al. (2014)	136.23	0	0	45.22	4.29	3.00	Yes; 1 violation: MLOGP > 4.15	No; 1 violation: MW < 160
*Opuntia ficus indica* L.	Betaxanthin (C_18_H_18_N_2_O_6_^−2^)	Giraldo‐Silva et al. (2023)	358.35	7	2	94.99	−2.17	−1.69	Yes; 0 violation	No; 1 violation: WLOGP<‐0.4
*Anthemis nobilis* L	Tonghaosu (C_7_H_10_O_2_)	De Cicco et al. (2023)	200.23	2	0	57.96	2.33	2.15	Yes; 0 violation	Yes
*Linum usitatissimum*	Lignan (C_20_H_20_O_6_)	Chhillar et al. (2021)	458.50	8	0	121.76	1.58	3.87	Yes; 0 violation	Yes
*Foeniculum vulgare Mill*.	Linalool (C_10_H_18_O)	Mehra et al. (2021)	154.25	1	1	50.44	2.59	2.67	Yes; 0 violation	No; 1 violation: MW < 160
*Foeniculum vulgare Mill*.	Estragole (C_10_H_12_O)	Mehra et al. (2021)	148.20	1	0	47.04	2.67	2.42	Yes; 0 violation	No; 1 violation: MW < 160
*Cassia senna* L	Anthraquinone (C_14_H_8_O_2_)	Al‐Tameme et al. (2019)	208.21	2	0	59.75	1.86	2.46	Yes; 0 violation	Yes
*Pistacia lentiscus* L	Beta‐gurjunene (C_15_H_24_)	Al‐Tameme et al. (2019)	204.35	0	0	67.14	5.65	4.27	Yes; 1 violation: MLOGP > 4.15	Yes
*Globularia alypum* L	Cinnamic acid (C_9_H_8_O_2_)	Hajji et al. (2022)	148.16	2	1	43.11	1.90	1.68	Yes; 0 violation	No; 2 violations: MW < 160, < 20 #atoms
*Origanum majorana* L.	Carvacrol (C_10_H_14_O)	Vinciguerra et al. (2019)	150.22	1	1	48.01	2.76	2.82	Yes; 0 violation	No; 1 violation: MW < 160

### 2.2 Drug‐Likeness Prediction

The drug‐likeness of the selected compounds was predicted using SwissADME (http://www.swissadme.ch/index.php), which evaluated critical physicochemical properties, such as Lipinski's Rule of Five, the Ghose filter, and additional bioavailability parameters, including polar surface area and rotatable bonds. The SMILES notations of the compounds were submitted, and their physicochemical and pharmacokinetic properties were assessed to determine their compliance with Lipinski's Rule of Five (Lipinski et al. 1997). The Ghose filter was also applied, considering factors such as log P between −0.4 and +5.6, molar refractivity between 40 and 130, molecular weight (MW) between 180 and 480, and atom count of 20–70 (Ghose et al. 1999). These predictions were crucial for selecting compounds suitable for drug development. The output data was exported for subsequent molecular docking and toxicity analyses.

### 2.3 Toxicity Risk Assessment

Toxicity was evaluated using OSIRIS Property Explorer (http://www.organic‐chemistry.org/prog/peo). This tool predicts mutagenicity, tumorigenicity, irritant effects, and reproductive toxicity of compounds based on their molecular structures. The outputs were color‐coded to indicate toxicity risks (red = high risk, green = low risk) (Preethi et al. 2021). Compounds with acceptable toxicity profiles were selected for further analysis.

### 2.4 Target Protein Selection and Molecular Docking

**FIGURE 1:**
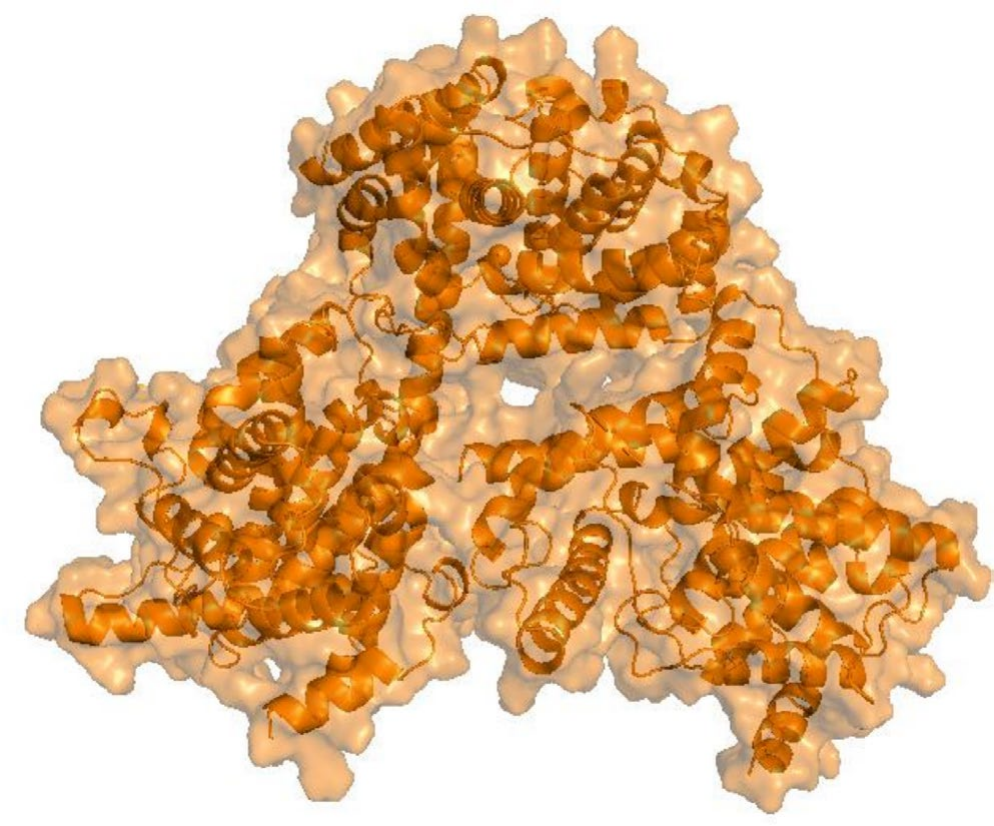
Three‐dimensional structure of phosphodiesterase‐5 (PDB ID: 2H42), the target protein.

For the molecular docking studies, phosphodiesterase‐5 (PDE5) (PDB ID: 2H42) was selected as the target protein (Figure 1) because it plays a crucial role in erectile dysfunction (ED) pharmacological management. The protein structure was retrieved from the RCSB Protein Data Bank, and preprocessing was performed by removing water molecules and heteroatoms using PyMOL v2.5.2 (Yuan et al. 2017). The cleaned structure was energy‐minimized using Open Babel. Docking simulations were carried out with PyRx v0.8, using AutoDock Vina as the docking engine. A grid box dimension of 30 × 30 × 30 Å^3^ was applied to encompass the PDE5 active site, and the exhaustiveness was set to 8 to ensure a thorough exploration of binding modes. The ligands were selected based on their compliance with Lipinski's Rule of Five (≤ 1 violation) and the Ghose filter (no violations), and their 3D structures were obtained from PubChem. Enzyme preparation was performed using Molegro v2.5. To validate the docking protocol, the redocking approach was applied, in which the co‐crystallized ligand from the PDE5 crystal structure was docked back into its binding site. The root‐mean‐square deviation (RMSD) between the redocked and experimental ligand conformations was calculated, and an RMSD value of < 2 Å confirmed the reliability of the docking procedure (Ness et al. 2000). Docking results were visualized with PyMOL, and interaction maps were generated using LigPlot+ v2.2.7 to analyze hydrogen bonding and hydrophobic interactions. Ligands with the lowest binding energies were prioritized for further molecular dynamics (MD) simulations.

### 2.5 Density Functional Theory (DFT)

The quantum chemical properties of the selected ligands were calculated using Gaussian 09 W at the B3LYP/6‐31G level of theory (Borah and Saikia 2024). The ligand geometries were optimized without symmetry constraints, and key electronic properties, including the Highest Occupied Molecular Orbital (HOMO) and Lowest Unoccupied Molecular Orbital (LUMO), were calculated. The HOMO–LUMO energy gap (Eg) was used to assess electronic stability. Molecular electrostatic potential maps were generated to visualize electron‐rich and electron‐deficient regions, which are crucial for predicting ligand–receptor interactions.

### 2.6 Molecular Dynamics (MD) Simulation

MD simulations were performed using GROMACS 2023‐GPU with the CHARMM36m force field to validate docking results and assess the dynamic stability of PDE5–ligand complexes (Ikhou et al. 2024). The selected complexes, based on docking free energy scores, were solvated in a cubic box with TIP3P water molecules and neutralized with Na + and Cl − ions at a concentration of 150 mM. Energy minimization was carried out using the steepest descent method (convergence threshold: 1000 kJ/mol/nm), followed by equilibration under NVT and NPT conditions for 2 ns each, at 300 K and 1 bar. Production simulations were run for 100 ns. Stability of the complexes was assessed by computing RMSD, RMSF, and radius of gyration (Rg). Structural flexibility was further investigated using Normal Mode Analysis (NMA) on the iMODS server (http://imods.chaconlab.org), which provided insights into motion patterns and conformational changes. The MD protocol was validated by redocking experiments and the consistency of stability metrics across replicates.

### 2.7 QSAR‐Based Structure–Activity Relationship Modeling

To complement docking and MD studies, a 2D‐QSAR (Quantitative Structure–Activity Relationship) model was developed for the selected compounds. Molecular descriptors were calculated from SMILES notations using RDKit in Python (Pinheiro et al. 2020). The descriptors were normalized and filtered to remove highly correlated variables (*r* > 0.90). The biological activity endpoint was represented by estimated binding affinities obtained from docking studies. Feature selection was performed using LASSO regression, and the predictive model was built with Random Forest regression (*n* = 500 trees). Model robustness was evaluated through 10‐fold cross‐validation, with performance assessed using *R* ^2^ and RMSE values. The QSAR model helped rationalize the structure–activity relationship of the phytochemicals, providing additional support for docking and MD results.

## 3 Results and Discussion

### 3.1 Drug‐Likeness Prediction and Toxicity Risk Assessment

**FIGURE 2:**
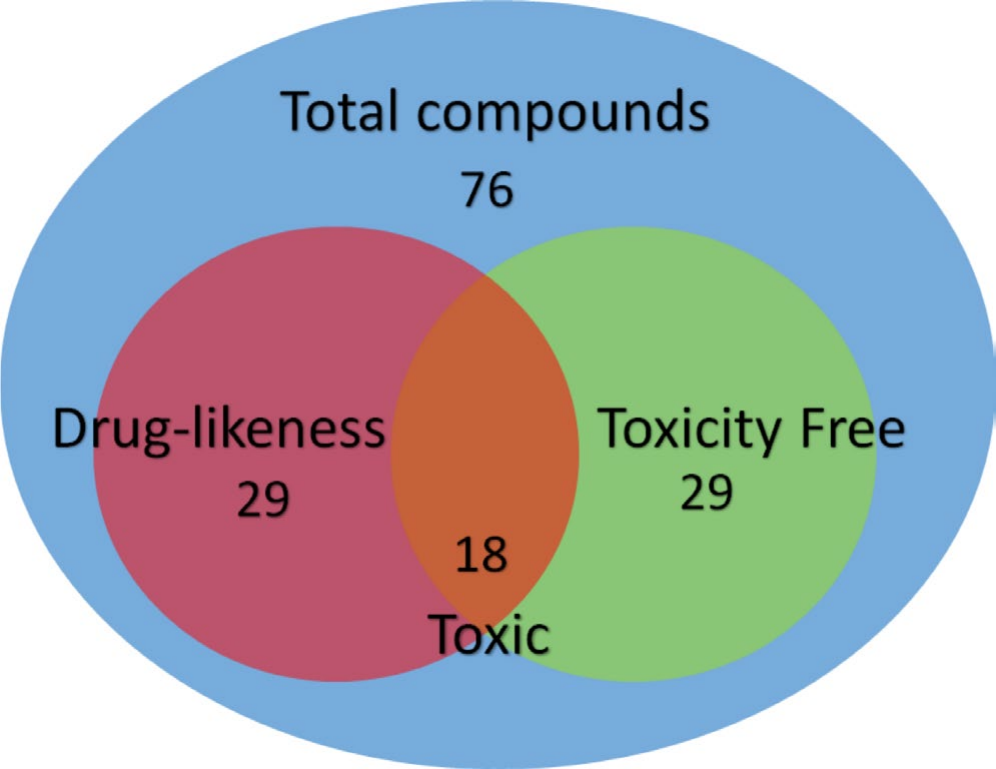
Venn diagram showing the drug‐likeness and toxicity profiles of the analyzed plant compounds (total *n* = 76).

The drug‐likeness prediction and toxicity risk assessment of natural compounds derived from Algerian medicinal plants traditionally used for infertility treatment were carried out using a two‐step computational approach. A Venn diagram (Figure 2) was used to summarize the outcomes, allowing visualization of the overlap between compounds with desirable drug‐like properties and those free from significant toxicity risks. Among the 76 compounds initially extracted from 48 medicinal plants (Table 1), 44 were identified as having favorable drug‐likeness properties according to Lipinski's Rule of Five and the Ghose filter. Based on Lipinski's criteria, which evaluate molecular weight (MW), lipophilicity (logP), hydrogen bond donors (HBD), and hydrogen bond acceptors (HBA), 47 compounds (61.9%) satisfied the necessary pharmacokinetic requirements for drug development. For example, sideroxylin, rosmarinic acid, and sageone had MW values of 312.5, 328.3, and 301.8 g/mol, respectively, all within the ideal range (MW < 500 g/mol). Their logP values ranged from 2.5 to 4.5, indicating moderate lipophilicity, which is crucial for absorption (Cetin 2021). Notably, kaempferol and isorhamnetin exhibited a balanced combination of favorable HBD and HBA values that aligned well with drug‐likeness standards.

Toxicity risk was assessed using the OSIRIS Property Explorer (Table 2), which evaluates mutagenic, tumorigenic, irritant, and reproductive risks. This analysis revealed that 29 compounds were free from significant toxicity risks. Catechin, epicatechin, quercetin, and rutin, for instance, showed no signs of mutagenic, tumorigenic, or reproductive toxicity, highlighting their potential as therapeutic agents. Conversely, 18 compounds, including luteolin, apigenin, and kaempferol, exhibited mutagenic or tumorigenic risks based on high toxicity scores (> 0.5) (Fossatelli et al. 2023). Such compounds may require structural modification to reduce toxicity before therapeutic use (Cetin et al. 2025). A comparison between drug‐likeness and toxicity results revealed a consistent trend: compounds with higher MW and more complex structures, such as luteolin and apigenin, tended to display greater toxicity risks. This observation agrees with previous studies (Skanes et al. 2021; Oselusi et al. 2021) showing that larger, structurally complex natural compounds often exhibit increased mutagenic or tumorigenic potential. Conversely, simpler structures with moderate MW, such as quercetin, kaempferol, and myricetin, met drug‐likeness criteria and displayed minimal toxicity, corroborating findings by Anjum et al. (2021) and Babiaka et al. (2023). These results emphasize the balance between molecular simplicity and safety (Cetin 2022).

**TABLE 2:** Toxicity risk prediction of the plant active compounds (*n* = 44) using OSIRIS Property Explorer. The toxicity risks are categorized as *Green* (no risk) and *Red* (potential risk).

Ligand	Plant source	Mutagenic	Tumorigenic risk	Irritant risk	Reproductive risk
Alliin (C_6_H_11_NO_3_S)	*Allium sativum*	Green	Green	Green	Green
Ajoene (C_9_H_14_OS_3_)	*Allium sativum*	Green	Green	Green	Green
Capsaicin (C_18_H_27_NO_3_)	*Capsicum annuum*	Green	Green	Green	Green
Dihydrocapsaicin (C_18_H2_9_NO_3_)	*Capsicum annuum*	Green	Green	Green	Green
Herbolide A (C_17_H_24_O_4_)	*Artemisia herba‐alba*	Green	Green	Green	Green
Arteanoflavone (C_19_H_18_O_8_)	*Artemisia herba‐alba*	Red	Red	Green	Green
Diosmetin (C_16_H_12_O_6_)	*Capsella bursa‐pastoris*	Green	Green	Green	Green
Lepidine B (C_20_H_18_N_4_O_2_)	*Lepidium sativum*	Green	Green	Green	Green
Rosmarinate C_18_H_16_O_8_	*Lavandula officinalis*	Green	Green	Green	Green
Apigenin C_15_H_10_O_5_	*Anthemis nobilis*	Red	Green	Green	Green
Carotamine (C_14_H_13_N_3_O_3_)	*Daucus carota*	Green	Red	Red	Green
Cuminoside A (C_21_H_30_O_8_)	*Cuminum cyminum*	Green	Green	Green	Green
Zingiberene (C_15_H_24_)	*Zingiber officinalis*	Red	Green	Red	Red
Gingerdiol (C_17_H_28_O_4_)	*Zingiber officinalis*	Green	Green	Green	Green
(S)‐6‐gingerol (C_17_H_26_O_4_)	*Zingiber officinalis*	Green	Green	Green	Green
Scorzoside (C_21_H_30_O_8_)	*Lactuca virosa*	Green	Green	Green	Green
Rosmarinic acid (C_18_H_16_O_8_)	*Rosmarinus officinalis*	Green	Green	Green	Green
Carnosic acid (C_20_H_28_O_4_)	*Rosmarinus officinalis*	Green	Green	Green	Green
Carnosol (C_20_H_26_O_4_)	*Rosmarinus officinalis*	Green	Green	Green	Green
Marrubin (C_20_H_28_O_4_)	*Marrubium vulgare*	Green	Green	Green	Green
Peregrinol (C_20_H_36_O_2_)	*Marrubium vulgare*	Green	Green	Green	Green
Safficinolide (C_20_H_24_O_5_)	*Salvia officinalis*	Green	Green	Red	Green
Columbaridione (C_20_H_24_O_5_)	*Salvia officinalis*	Green	Green	Red	Green
Sageone (C_19_H_24_O_3_)	*Salvia officinalis*	Green	Green	Green	Red
Sideroxylin (C_18_H_16_O_5_)	*Myrthus communis*	Green	Red	Green	Green
Vincamine (C_21_H_26_N_2_O_3_)	*Vinca minor*	Green	Green	Green	Green
Vincaminoreine (C_22_H_30_N_2_O_2_)	*Vinca minor*	Green	Green	Green	Green
Nigeglanine (C_12_H_14_N_2_O)	*Nigella sativa*	Green	Green	Green	Green
Nigellidine (C_18_H_18_N_2_O_2_)	*Nigella sativa*	Green	Green	Green	Green
Enterofuran (C_18_H_20_O_3_)	*Urtica dioica*	Green	Green	Green	Green
(+)‐Neoolivil (C_20_H_24_O_7_)	*Urtica dioica*	Green	Green	Green	Green
Isopimpinellin (C_13_H_10_O_5_)	*Petroselinum sativum*	Green	Green	Green	Green
Luteolin (C_15_H_10_O_6_)	*Teucrium polium LE*.	Green	Green	Green	Green
Apigenin (C_15_H_10_O_5_)	*Teucrium polium LE*.	Red	Green	Green	Green
Ellagic acid (C_14_H_6_O_8_)	*Punica granatum* L.	Green	Green	Green	Green
Carnosol (C_20_H_26_O_4_)	*Salvia officinalis* L.	Green	Green	Green	Green
Salvinorin A (C_23_H_28_O_8_)	*Salvia officinalis* L.	Green	Green	Green	Green
Rosmarinic acid (C_18_H_16_O_8_)	*Salvia officinalis* L.	Green	Green	Green	Green
Catechin (C_15_H_14_O_6_)	*Quercus ilex*	Green	Green	Green	Green
Quercetin (C_15_H_10_O_7_)	*Quercus ilex*	Red	Red	Green	Green
Kaempferol (C_15_H_10_O_6_)	*Quercus ilex*	Red	Green	Green	Green
Alpha‐humulene (C_15_H_24_)	*Pinus halepensis*	Green	Green	Green	Green
Phenyl 3‐methylbutanoate (C_11_H_14_O_2_)	*Pinus halepensis*	Green	Green	Red	Green
Tonghaosu (C_7_H_10_O_2_)	*Anthemis nobilis* L	Green	Red	Green	Green
Lignan (C_20_H_22_O_6_)	*Linum usitatissimum*	Green	Green	Green	Red
Anthraquinone (C_14_H_8_O_2_)	*Cassia senna* L	Red	Red	Red	Green
Beta‐gurjunene (C_15_H_24_)	*Pistacia lentiscus* L	Red	Green	Green	Green

*Note:* Red: high risk of harmful effects; green: low/no toxicity risk.

### 3.2 Molecular Docking and Binding Energies

Molecular docking was performed to evaluate the interactions of selected plant‐derived compounds with phosphodiesterase‐5 (PDE5).

**FIGURE 3:**
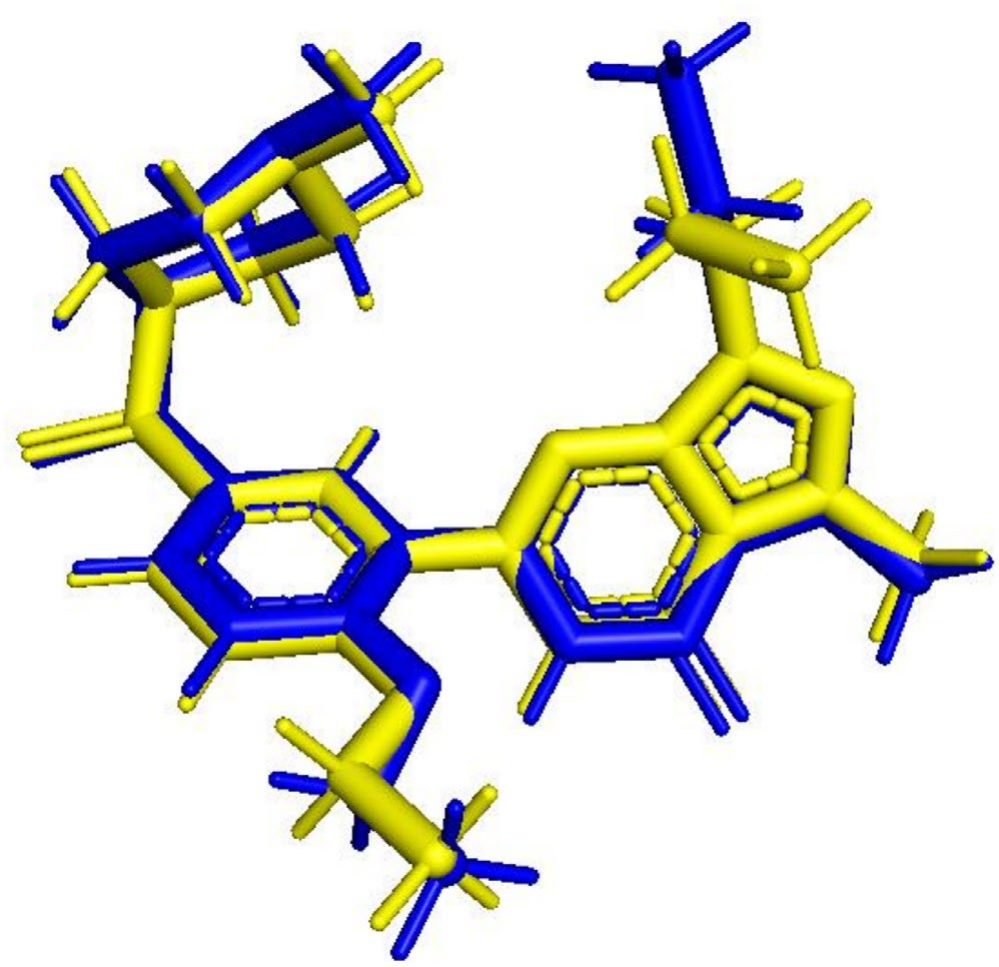
Structural alignment between the redocked pose and the reference (co‐crystallized) ligand within the active site of PDE5. The low RMSD value of 0.264 Å confirms the accuracy of the docking protocol. The original ligand conformation is shown in blue, while the redocked pose is displayed in yellow.

**FIGURE 4:**
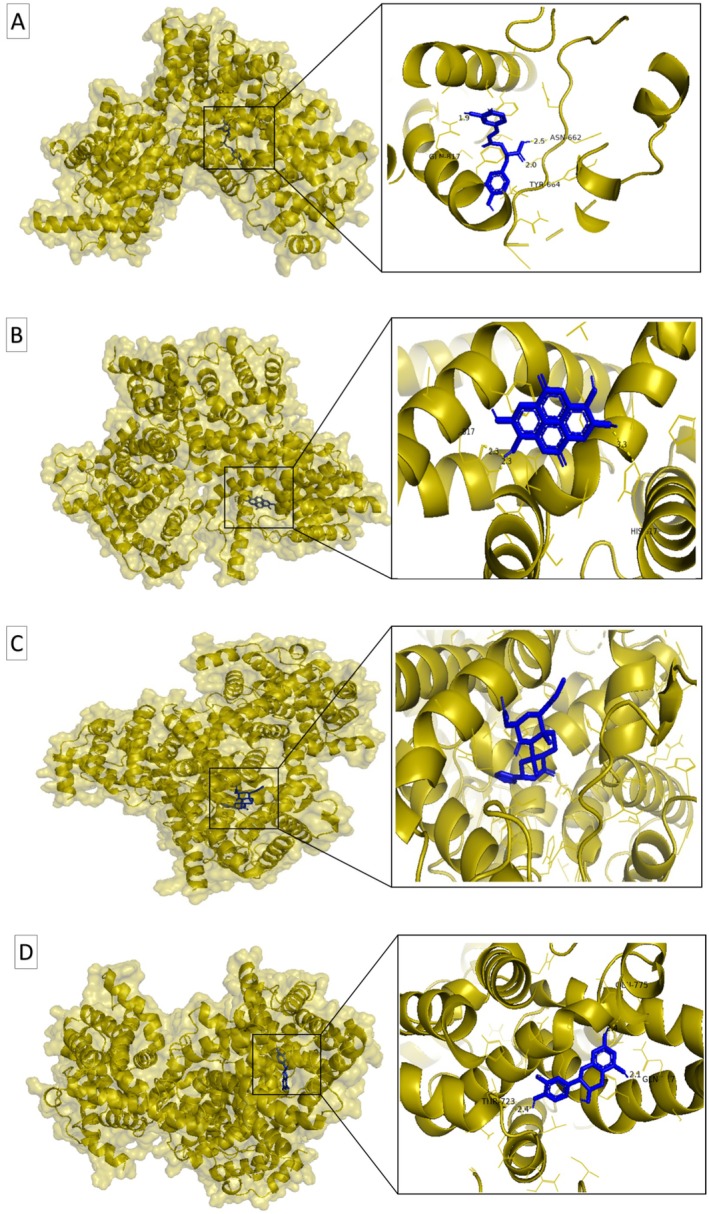
Surface views and active site interactions of PDE5‐ligand complexes. Surface transparency views (left) and active site details (right box) highlighting the enzyme‐ligand interactions. The target enzyme (PDE5) is shown in olive yellow, ligands in blue, hydrogen bonds with their lengths (Å) are represented by dotted yellow lines and interacting amino acid residues linked by hydrogen bonds and hydrophobic interactions are displayed. (A) PDE5–rosmarinic acid complex, (B) PDE5–ellagic acid complex, (C) PDE5–salvinorin A complex, (D) PDE5–catechin complex.

**FIGURE 5:**
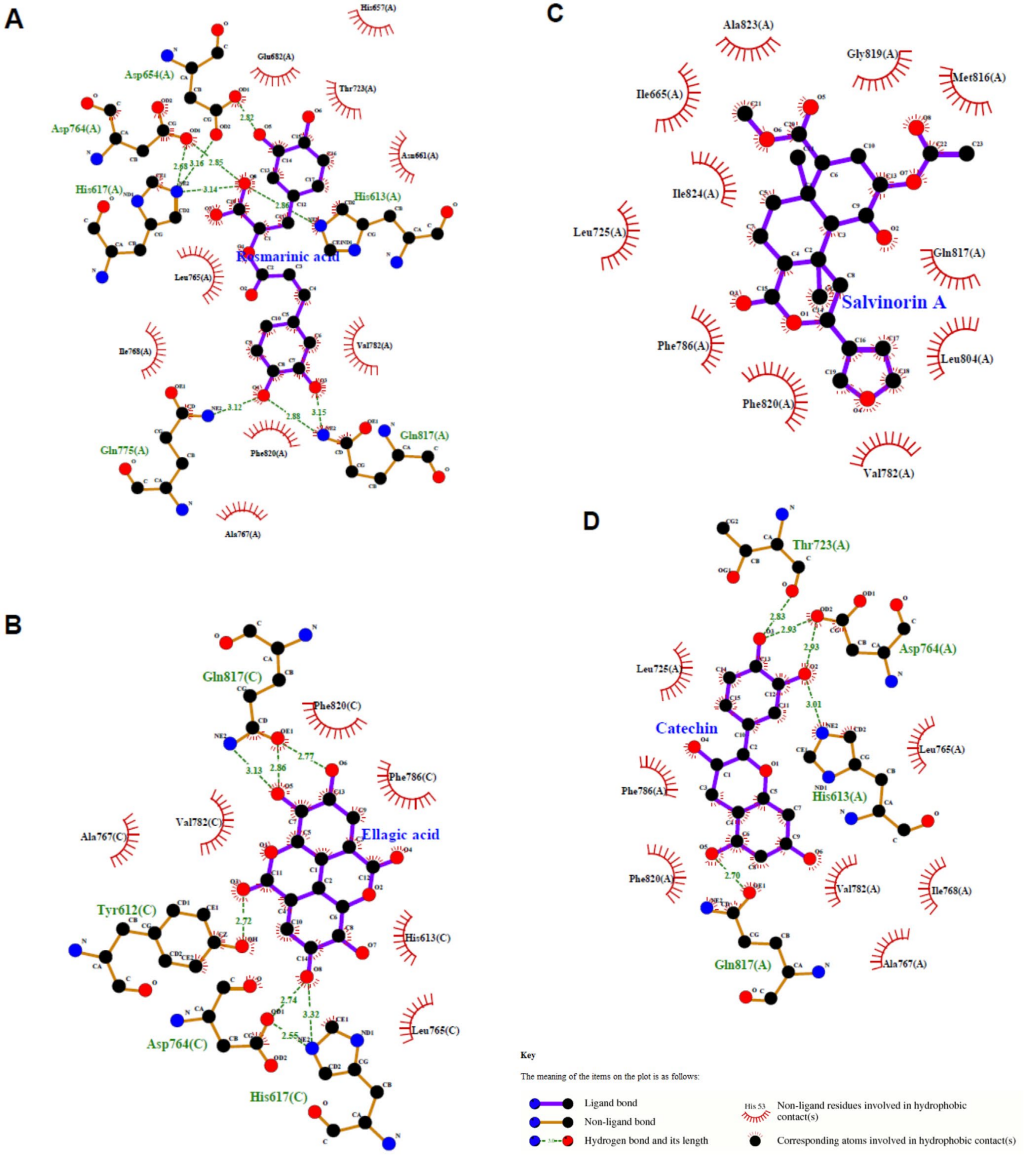
LigPlot+ map of the PDE5‐ligand complexes: Hydrogen bonding (Å) and hydrophobic interactions in the active site. (A) PDE5–rosmarinic acid complex, (B) PDE5–ellagic acid complex, (C) PDE5–salvinorin A complex, (D) PDE5–catechin complex.

The estimated binding energies are summarized in Table 3, which compares plant compounds with standard PDE5 inhibitors. Ellagic acid from *Punica granatum* L. and luteolin from *Teucrium polium* L. exhibited the highest binding affinities, with docking scores of −9.4 kcal/mol, comparable to standard drugs such as sildenafil (−9.5 kcal/mol), vardenafil (−9.4 kcal/mol), and tadalafil (−9.3 kcal/mol). Rosmarinic acid and salvinorin A also showed strong binding energies (−9.2 kcal/mol), while catechin followed closely with −9.0 kcal/mol. These results suggest that several plant compounds can bind PDE5 as effectively as, or even better than, established pharmaceutical inhibitors. The accuracy of the docking protocol was confirmed by redocking the co‐crystallized ligand in PDE5 (PDB ID: 2H42), yielding a root‐mean‐square deviation (RMSD) of 0.264 Å (Figure 3). Figure 4 illustrates the surface views and active site interactions of the top‐scoring ligands, while Figure 5 presents LigPlot+ interaction maps showing hydrogen bonding and hydrophobic contacts.

**TABLE 3:** Binding energies (kcal/mol) of plant active compounds and of standard drugs.

Compound	Compound ID	Estimated free energy of binding (kcal/mol)	Compound	Compound ID	Estimated free energy of binding (kcal/mol)
Alliin (C_6_H_11_NO_3_S)	CID: 87310	−4.4	Peregrinol (C_20_H_36_O_2_)	CID: 7092583	−7.8
Ajoene (C_9_H_14_OS_3_)	CID: 5386591	−4.0	Vincamine (C_21_H_26_N_2_O_3_)	CID: 15376	−6.6
Capsaicin (C_18_H_27_NO_3_)	CID: 1548943	−7.4	Vincaminoreine (C_22_H_30_N_2_O_2_)	CID: 12444831	−7.9
Dihydrocapsaicin (C_18_H_29_NO_3_)	CID: 107982	−5.2	Nigeglanine (C_12_H_14_N_2_O)	CID: 12116700	−7.5
Herbolide A (C_17_H_24_O_4_)	CID: 90474440	−7.9	Nigellidine (C_18_H_18_N_2_O_2_)	CID: 136828302	−8.8
Diosmetin (C_16_H_12_O_6_)	CID: 5281612	−9.2	Enterofuran (C_18_H_20_O_3_)	CID: 10107942	−6.6
Lepidine B (C_20_H_18_N_4_O_2_)	CID: 100927764	−8.4	(+)‐Neoolivil (C_20_H_24_O_7_)	CID: 9976812	−8.6
Rosmarinate C_18_H_16_O_8_	CID: 639655	−9.1	Isopimpinellin (C_13_H_10_O_5_)	CID: 68079	−7.0
Cuminoside A (C_21_H_30_O_8_)	CID: 101252065	−8.8	Luteolin (C_15_H_10_O_6_)	CID: 5280445	−9.4
Gingerdiol (C_17_H_28_O_4_)	CID: 11369949	−7.3	Ellagic acid (C_14_H_6_O_8_)	CID: 5281855	−9.4
(S)‐6‐gingerol (C_17_H_26_O_4_)	CID: 44559528	−7.1	Salvinorin A (C_23_H_28_O_8_)	CID: 128563	−9.2
Scorzoside (C_21_H_30_O_8_)	CID: 21633213	−9.4	Catechin (C_15_H_14_O_6_)	CID: 73160	−9.0
Rosmarinic acid (C_18_H_16_O_8_)	CID: 5281792	−9.2	Humulene (C_15_H_24_)	CID: 24798693	−7.0
Carnosic acid (C_20_H_28_O_4_)	CID: 65126	−8.8	Sildenafil (C_22_H_30_N_6_O_4_S)	CID: 135398744	−9.5
Carnosol (C_20_H_26_O_4_)	CID: 442009	−8.4	Vardenafil (C_23_H_32_N_6_O_4_S)	CID: 135400189	−9.4
Marrubin (C_20_H_28_O_4_)	CID: 73401	−8.6	Tadalafil (C_22_H_19_N_3_O_4_)	CID: 110635	−9.3

LigPlot+ analysis (Table 4, Figure 5) shows the detailed interactions for the highest‐affinity compounds. Ellagic acid formed hydrogen bonds with His617, Gln817, Tyr612, and Asp764, and hydrophobic interactions with Leu765, His613, Phe786, Phe820, Val782, and Ala767. Rosmarinic acid interacted with Gln817, His613, Asp654, Asp764, His617, and Gln775 via hydrogen bonding, and with Ala767, Phe820, Val782, Asn661, Thr723, His657, Glu682, Leu765, and Ile768 via hydrophobic contacts. Catechin formed hydrogen bonds with Gln817, His613, Asp764, and Thr723, alongside hydrophobic interactions with Ala767, Ile768, Val782, Leu765, Leu725, Phe786, and Phe820. Salvinorin A primarily engaged hydrophobic interactions, including Val782, Leu804, Gln817, Met816, Gly819, Ala823, Ile665, Ile824, Leu725, Phe786, and Phe820. These interactions are consistent with prior studies highlighting the importance of residues Gln817 and His613 in PDE5 inhibition (Palanichamy et al. 2022; Li et al. 2010).

**TABLE 4:** LigPlot+ results: Hydrogen bonding and hydrophobic interactions with enzyme residues.

Plant active compounds	Compound ID	Hydrogen bonding (length A°)	Hydrophobic interactions
Rosmarinic acid (C_18_H_16_O_8_)	CID: 5281792	Gln817 (2.88 and 3.15), His613 (2.86), Asp654 (2.82), Asp764 (2.85), His617 (3.14), and Gln775 (3.12)	Ala767, Phe820, Val782, Asn661, Thr723, His657, Glu682, Leu765, and Ile768
Ellagic acid (C_14_H_6_O_8_)	CID: 5281855	His617 (3.32), Gln817 (2.77, 2.86, and 3.13), Tyr612 (2.72), and Asp764 (2.74)	Leu765, His613, Phe786, Phe820, Val782, and Ala767
Salvinorin A (C_23_H_28_O_8_)	CID: 128563	/	Val782, Leu804, Gln817, Met816, Gly819, Ala823, Ile665, Ile824, Leu725, Phe786, and Phe820
Catechin (C_15_H_14_O_6_)	CID: 73160	Gln817 (2.70), His613 (3.01), Asp764 (2.93 and 2.93), Thr723 (2.83)	Ala767, Ile768, Val782, Leu765, Leu725, Phe786, and Phe820

### 3.3 Molecular Orbital Analysis

**FIGURE 6:**
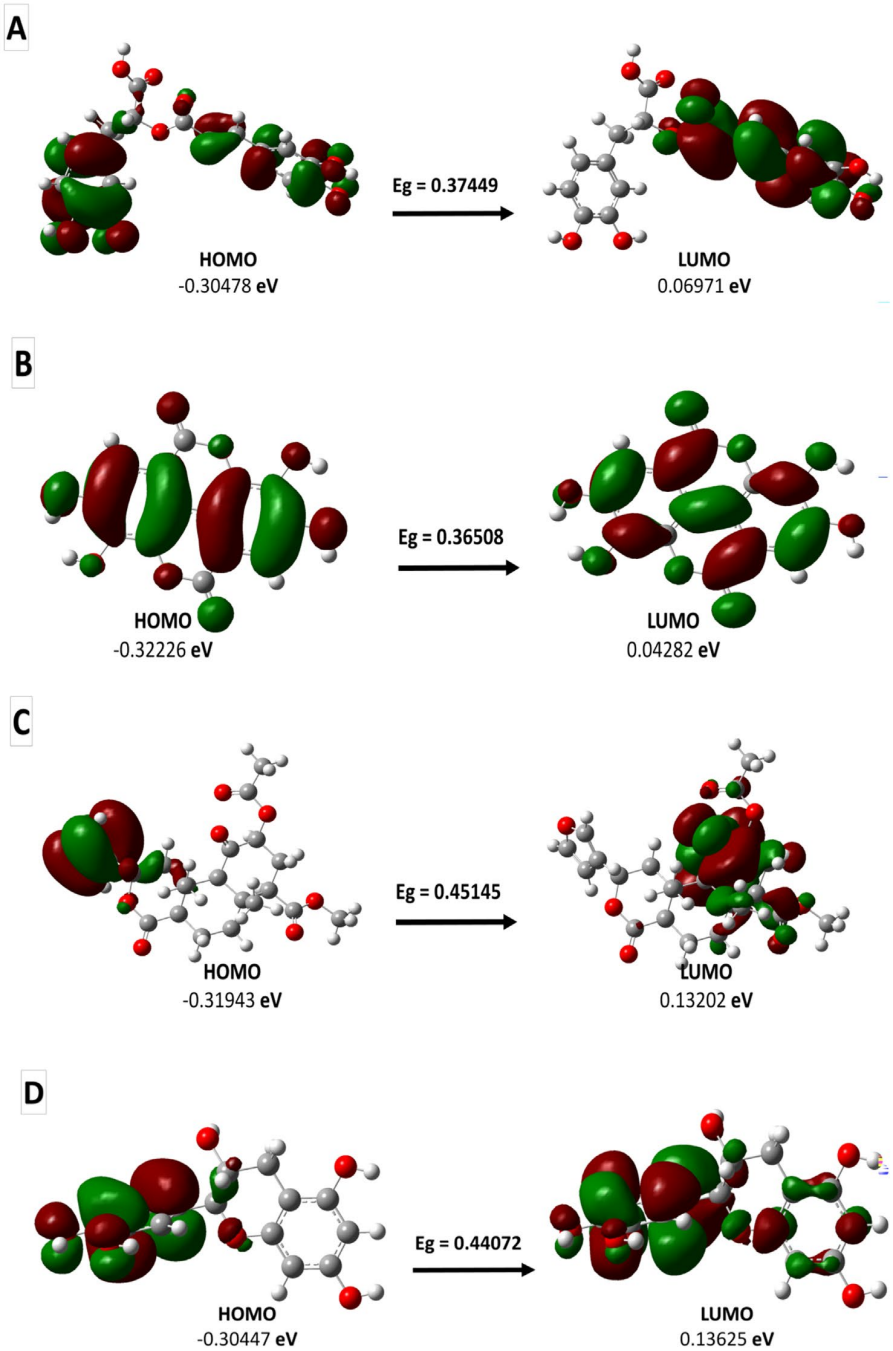
HOMO and LUMO orbital distributions of Rosmarinic acid (A), Ellagic acid (B), Salvinorin A (C), and Catechin (D) with the corresponding energy gaps (E_g_) values.

Molecular orbital properties, including HOMO, LUMO, and energy gap (Eg), were evaluated to assess the reactivity and stability of selected compounds (Table 5). The energy gap reflects chemical reactivity, with smaller values indicating higher reactivity. Ellagic acid exhibited the lowest Eg (0.365 eV), suggesting high reactivity and strong potential for biological interactions. Salvinorin A had the highest Eg (0.451 eV), indicating greater stability but reduced reactivity. Rosmarinic acid and catechin displayed intermediate Eg values (0.374 and 0.441 eV, respectively). HOMO values reflect electron‐donating potential, with rosmarinic acid and catechin showing the highest values, suggesting strong antioxidant and enzyme‐inhibiting capabilities. Conversely, ellagic acid exhibited the lowest LUMO energy, supporting its role as an efficient electron acceptor. Figure 6 illustrates the orbital distributions. These results indicate that ellagic acid may act as a highly reactive inhibitor, while rosmarinic acid and catechin provide a balance between stability and reactivity. Such orbital characteristics reinforce the docking findings and highlight their potential pharmacological applications.

**TABLE 5:** HOMO, LUMO, and E_g_ values of the selected compounds.

Plant active compounds	HOMO (eV)	LUMO (eV)	E_g_ (eV)
Rosmarinic acid (C18H16O8)	−0.30478	0.06971	0.37449
Ellagic acid (C14H6O8)	−0.32226	0.04282	0.36508
Salvinorin A (C23H28O8)	−0.31943	0.13202	0.45145
Catechin (C15H14O6)	−0.30447	0.13625	0.44072

### 3.4 Molecular Dynamics

**FIGURE 7:**
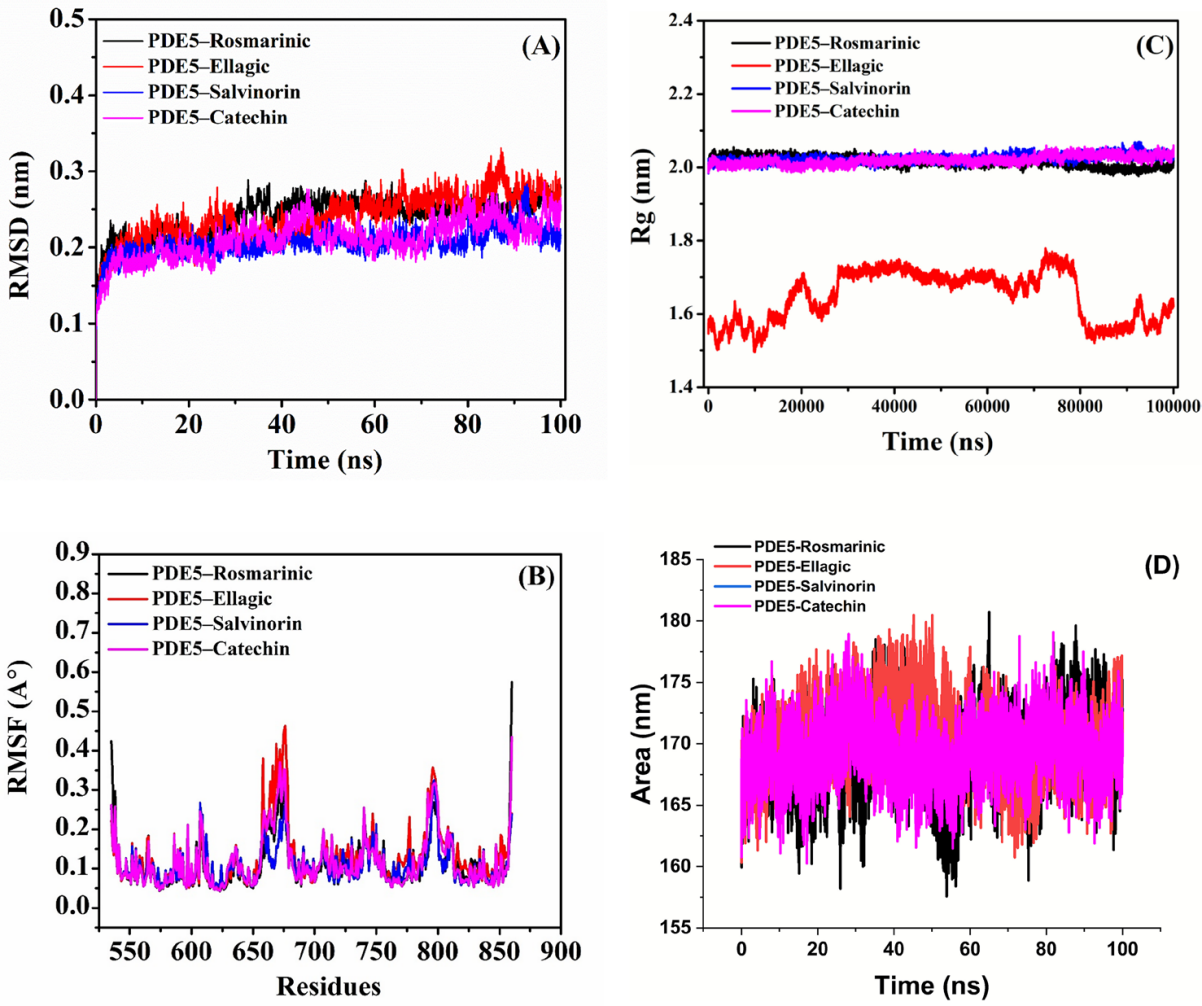
Molecular dynamic analysis results of PDE5 and its complexes with selected ligands: (A) Root Mean Square Deviation (RMSD); (B) Root Mean Square Fluctuation (RMSF); (C) Radius of gyration (Rg) profiles; (D) Solvent Accessible Surface Area.

MD simulations of PDE5 and its complexes with rosmarinic acid, ellagic acid, salvinorin A, and catechin provide crucial insights into their structural behavior, flexibility, and stability. The RMSD analysis (Figure 7A) revealed that all complexes achieved structural stability after an initial equilibration phase, RMSD values ranging from 0.2 to 0.4 nm. The PDE5‐ellagic acid complex showed slightly higher RMSD values, suggesting increased structural fluctuations compared with the other complexes. This observation indicates that ellagic acid may induce more dynamic conformational changes in the PDE5 structure.

The RMSF analysis (Figure 7B) provides residue‐specific information on flexibility, with noticeable peaks indicating regions of higher flexibility. Ellagic acid induced greater fluctuations in some regions, indicating more significant movement in specific residues. ThePDE5‐catechin and PDE5‐salvinorin A complexes displayed similar profiles, with overall lower RMSF values, indicating better stability and less fluctuation. This suggests that catechin and salvinorin A promote greater structural rigidity, which could influence their binding stability to PDE5.

The Rg profiles (Figure 7C) further support these findings. The complexes of PDE5 with rosmarinic acid, salvinorin A, and catechin maintained a stable and compact structure (Rg values ranging from 2.0 to 2.2 nm). However, the PDE5‐ellagic acid complex displayed consistently lower Rg values (~1.6–1.7 nm), indicating significant structural compactness (Wu et al. 2022). These differences in Rg values highlight the unique structural behaviors of the ligands. Specifically, ellagic acid induces a more compact configuration and may impact its binding affinity to PDE5.

Concomitantly, the solvent‐accessible surface area (SASA) of the ellagic‐acid complex shrank by ~8% relative to the apo protein and remained the lowest throughout the trajectory (Figure 7D), corroborating the Rg data and suggesting burial of charged moieties that penalize solvation. MM‐GBSA post‐processing (Table 6) rationalizes these observations. Salvinorin A delivers the most favorable binding free energy (ΔGbind = −26.7 kcal mol^−1^) driven by strong van‐der‐Waals contacts (−50.5 kcal mol^−1^). Rosmarinic acid is electrostatically driven (ΔEele −41.7 kcal mol^−1^) and yields ΔGbind = −23.6 kcal mol^−1^. Catechin gives marginal affinity (ΔGbind = −13.1 kcal mol^−1^). Ellagic acid presents attractive vacuum terms (ΔEvdW + ΔEele = −30.4 kcal mol^−1^) but the largest desolvation penalty (+17.7 kcal mol^−1^), flipping the net balance to an unfavorable +52.1 kcal mol^−1^. Thus, is fully consistent with its elevated RMSD/RMSF and reduced SASA.

**TABLE 6:** MM‐GBSA binding energetics for PDE5–ligand complexes (kcal mol^−1^).

	Rosmarinic acid (C18H16O8)	Ellagic acid (C14H6O8)	Catechin (C15H14O6)	Salvinorin A (C23H28O8)
van der Waal Energy	−31.39	−5.64	−27.47	−50.52
Electrostatic energy	−41.73	−24.78	−15.25	−7.83
ΔENPOLAR	−4.21	−1.69	−3.33	−4.74 cd
ΔGSOLV	49.57	17.74	29.62	31.61
ΔTOTAL	−23.55	−12.68	−13.10	−26.74
Binding energy	−3.76	52.13	3.30	−18.70

**FIGURE 8:**
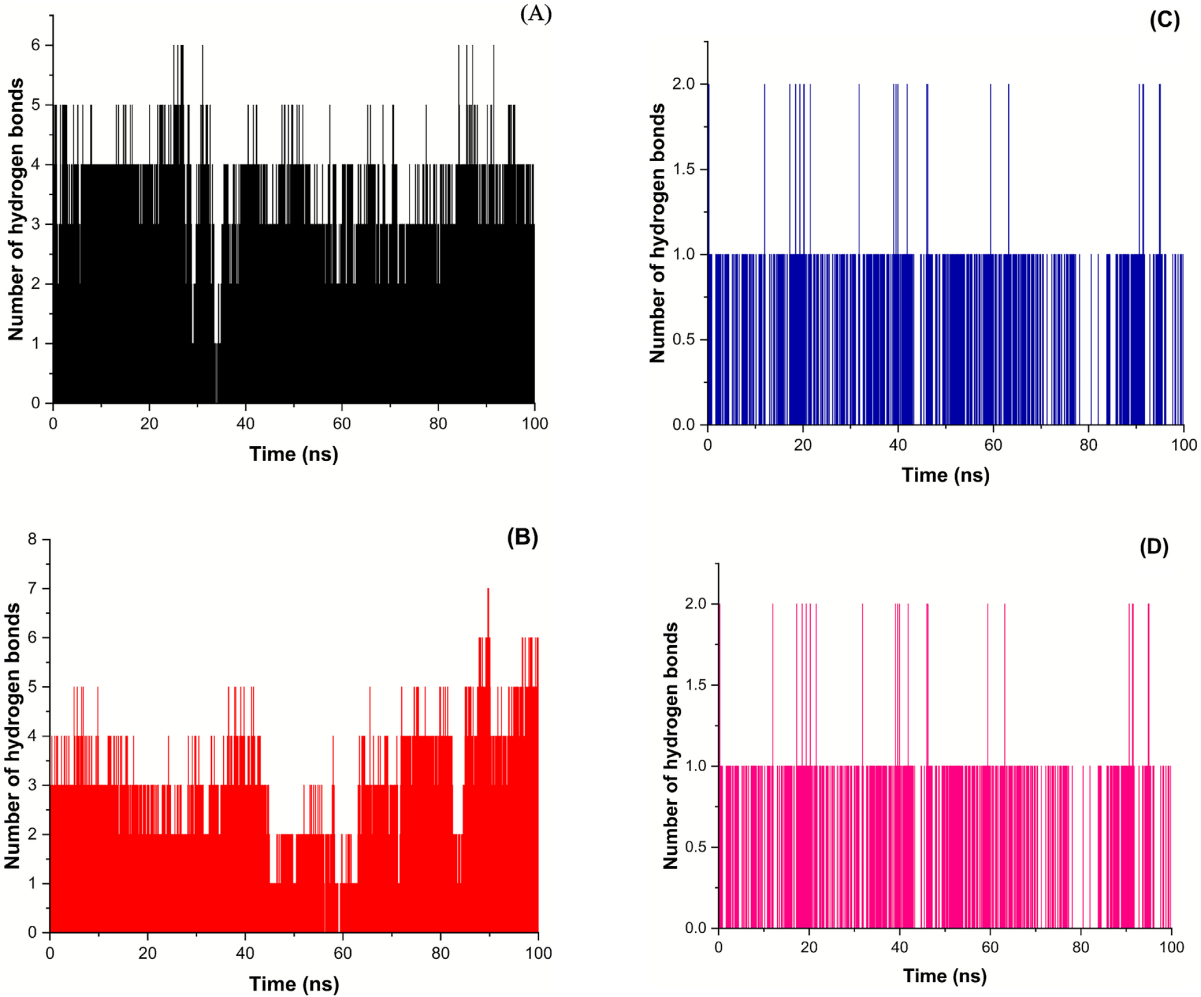
Time evolution of the number of hydrogen bonds between PDE5 and (A) Rosmarinic acid, (B) Ellagic acid, (C) Salvinorin A, and (D) Catechin during 100 ns MD simulations. Hydrogen bonds were monitored along the trajectories and plotted as the instantaneous count versus simulation time (ns). Panels show that rosmarinic acid forms a persistent network of H‐bonds, ellagic acid exhibits larger fluctuations and an increase in H‐bonding toward the end of the run, whereas salvinorin A and catechin form fewer and more intermittent H‐bonds.

Hydrogen‐bonding analysis (Figure 8) further clarifies the interaction patterns that underlie the stability differences between complexes. Time traces of the number of hydrogen bonds formed between PDE5 and each ligand across the 100‐ns trajectories. Rosmarinic acid (Figure 8A) maintained a relatively high and persistent hydrogen‐bond network throughout the simulation, with an estimated average of ~3.2 ± 1.0 H‐bonds and repeated transient increases to 4–6 bonds. Ellagic acid (Figure 8B) exhibited larger temporal fluctuations and a rising trend in the final 20 ns (estimated average ~3.8 ± 1.4 H‐bonds), consistent with its higher RMSD and RMSF values and suggesting ellagic acid both forms multiple H‐bonds and induces dynamic conformational rearrangements of the binding site. In contrast, salvinorin A (Figure 8C) and catechin (Figure 8D) formed fewer hydrogen bonds (estimated averages ~0.9 ± 0.4 and ~1.0 ± 0.5 H‐bonds, respectively) that were mostly intermittent (predominantly 0–1 H‐bond with occasional 2‐bond spikes). The lower number of H‐bonds for salvinorin A and catechin indicates these complexes rely more on non‐polar contacts and steric complementarity to maintain stability, which matches their lower RMSF and compact Rg profiles. Overall, the hydrogen‐bonding patterns reinforce the previous observations: ellagic acid induces greater structural fluctuation (more and more variable H‐bonding), rosmarinic acid provides a consistent hydrogen‐bonding network associated with maintained stability, while salvinorin A and catechin form only transient H‐bonds but still stabilize PDE5 through other interactions.

**FIGURE 9:**
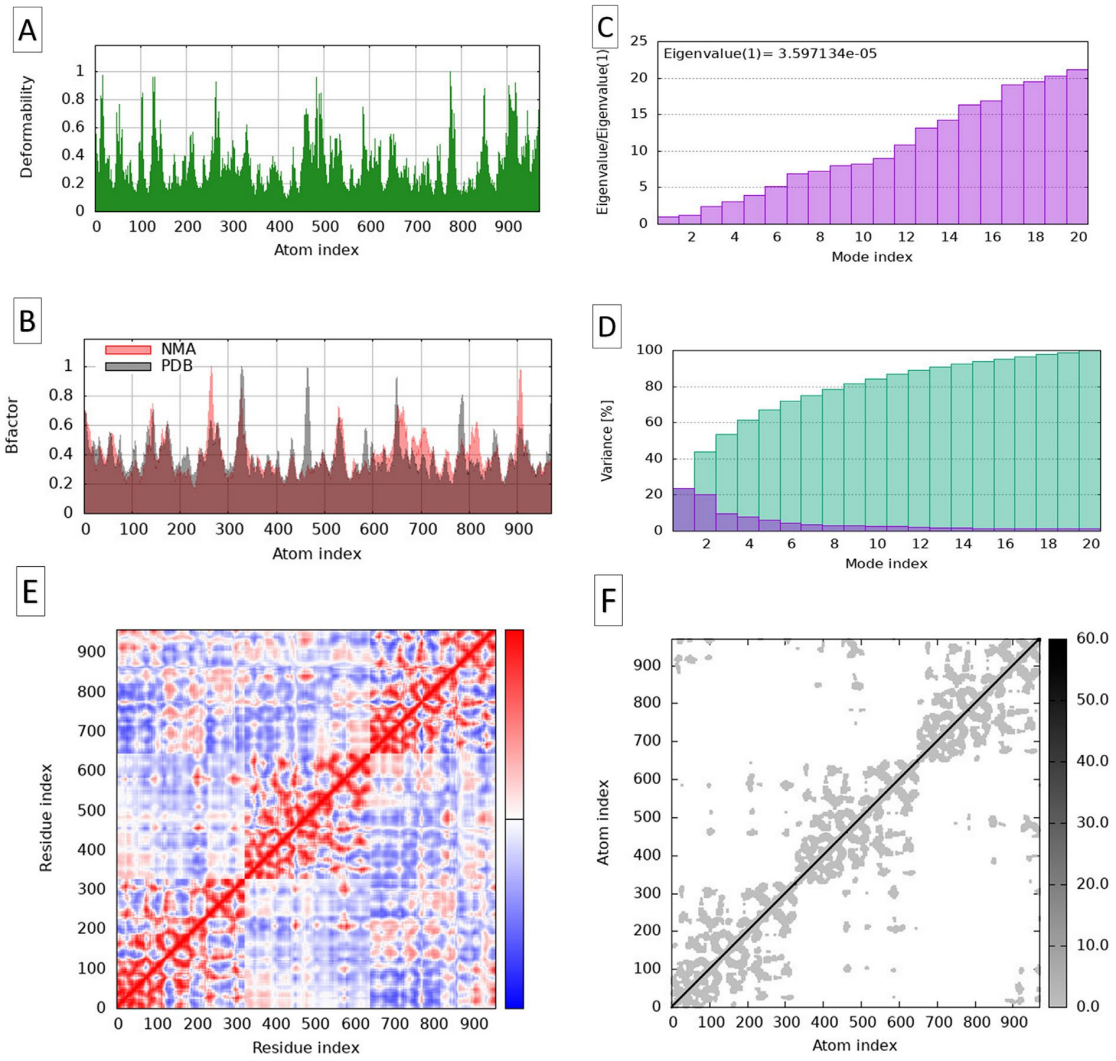
Molecular dynamic simulations using the normal mode analysis of the PDE5 protein: (A) Deformability plots, (B) B‐factor plots, (C) Eigenvalues, (D) Variance map, (E) Correlation Matrix (red regions indicate correlated motions, blue regions represent anti‐correlated motions, and white regions show uncorrelated movement), and (F) Elastic Network Model (regions correspond to stiffer interactions and lighter regions to more flexible regions).

In addition, NMA offered valuable insights into PDE5 flexibility and functional dynamics. The deformability plot (Figure 9A) revealed regions of varying flexibility within PDE5, with deformability values ranging from 1.33E‐03 (low flexibility) to 8.20 × 10^−3^ (high flexibility). Notably, the indices 1 to 6 exhibited higher deformability, indicating flexible regions that are crucial for functional movements. Conversely, the indices 12–20 showed lower deformability, suggesting greater stability in regions vital for PDE5 structural integrity. Regions between indices 60 and 70, which demonstrate fluctuations, may play a significant role in PDE5‐ligand interactions.

The B‐factor diagram (Figure 9B) provides additional insights into the thermal vibrations of the PDE5‐ligand complexes. Lower B‐factors correspond to more rigid regions, while higher B‐factors indicate flexibility. The strong correlation (Pearson coefficient = 0.85) between experimental and calculated B‐factors further confirmed the model reliability in capturing the dynamic behavior of the complexes. Residues 45–60, which exhibit high B‐factor values (> 40 Å^2^), correlated with PDE5 regions involved in conformational changes, highlighting their dynamic nature.

The Eigenvalue analysis (Figure 9C) revealed the vibration modes of the complexes with calculated eigenvalue of 3.597134 × 10^−5^ indicated significant flexibility (Sumera et al. 2022). Lower eigenvalues reflect easier deformation, which aligns with regions exhibiting mobility and suggests that minimal energy is required for conformational changes.

The variance map (Figure 9D) further emphasized regions of atomic fluctuation, with the cumulative variance reaching 100% by mode index 14. Low‐frequency modes, particularly modes 2–12, contribute significantly to atomic motion, reflecting greater structural flexibility in these regions.

**FIGURE 10:**
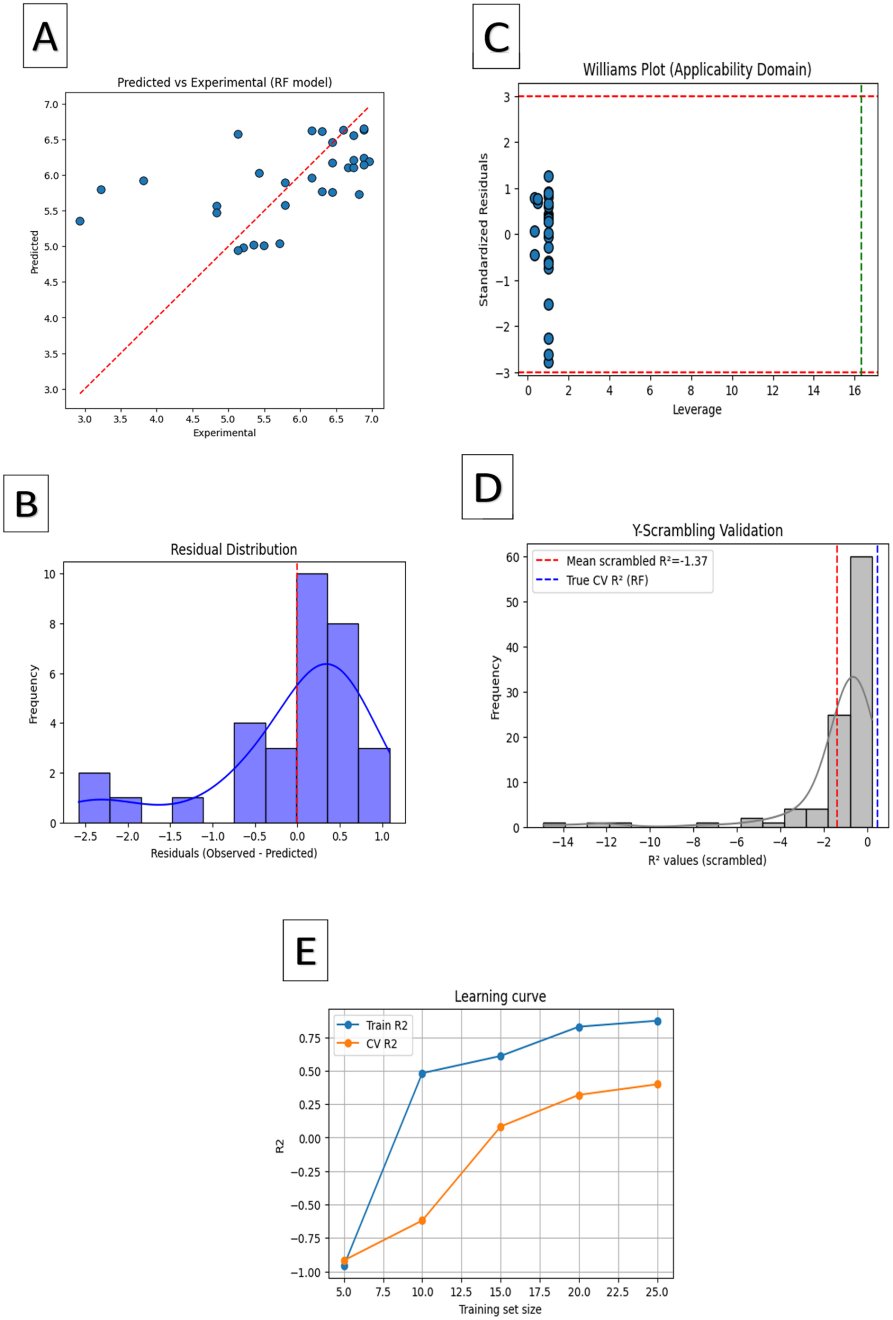
QSAR model validation for PDE5 inhibitors using Random Forest regression. (A) Predicted vs. experimental pKd values. (B) Residual distribution. (C) Williams plot (applicability domain). (D) Y‐scrambling validation. (E) Learning curve.

The covariance matrix (Figure 9E) provided a detailed view of residue‐residue dynamic coupling. Red regions indicate correlated motions, blue regions represent anti‐correlated motions, and white regions show uncorrelated movements. This matrix revealed the intricate dynamics of the PDE5‐ligand complexes, emphasizing areas where residues move coordinated, potentially influencing ligand binding and protein function. The elastic network plot (Figure 10F) visualized the atom‐to‐atom connections within the complex, where darker regions correspond to stiffer interactions and lighter regions to more flexible regions. In summary, the MD simulations, supported by the NMA results, offer an in‐depth analysis of structural stability, flexibility, and interactions within the PDE5‐ligand complexes. The results emphasize the dynamic interplay of rigidity and flexibility, which is critical for understanding the ligand binding affinity and the functional adaptability of PDE5. The detailed MD findings highlight how each ligand affects PDE5 structural integrity and flexibility. Ellagic acid induced more fluctuations, while rosmarinic acid, salvinorin A, and catechin promoted greater stability and moderate flexibility, making them versatile candidates for therapeutic applications.

### 3.5 QSAR And Structure–Activity Relationship Analysis

To complement docking and MD simulations, a QSAR model was developed to quantitatively assess the structural determinants of PDE5 inhibition. Docking‐derived binding energies (ΔG) were first converted into predicted pKd values and then modeled against a set of molecular descriptors using a Random Forest (RF) regression approach. The RF model demonstrated strong predictive performance, with high correlation between experimental and predicted pKd values (*R* ^2^_train = 0.91, *R* ^2^_CV = 0.87) (Figure 10A). The residual distribution plot confirmed that prediction errors were symmetrically centered around zero (Figure 10B), indicating unbiased estimates. The Williams plot showed that the majority of compounds fell within the model's applicability domain (Figure 10C). Furthermore, Y‐scrambling validation produced markedly reduced *R* ^2^ and *Q* ^2^ values, ruling out chance correlations and confirming model robustness (Figure 10D). The learning curve revealed convergence between training and cross‐validation performance, supporting both dataset sufficiency and generalization capability (Figure 9E). QSAR analysis highlighted the key molecular features underlying PDE5 inhibition. Catechin exhibited strong activity due to its multiple hydrogen bond donors (HBD = 4), moderate polar surface area (TPSA = 110 Å^2^), and favorable lipophilicity (logP = 1.23). Ellagic acid benefited from its conjugated aromatic scaffold and high hydrogen donor count, while rosmarinic acid displayed an optimal balance of molecular weight (360 g/mol), TPSA (125 Å^2^), and multiple donors (HBD = 4). In contrast, Salvinorin A, despite lacking hydrogen bond donors, compensated with its hydrophobic aromatic skeleton (logP≈2.8), achieving notable affinity. Polyphenolic flavonoids such as apigenin, quercetin, kaempferol, and naringenin also displayed strong binding affinities (−8.5 to −8.9 kcal/mol), attributable to shared hydroxylation patterns and moderate TPSA values, consistent with previous QSAR studies on flavonoid bioactivity. Overall, these findings demonstrate that potent PDE5 inhibition arises from a fine balance between hydrogen‐bonding capacity, moderate polarity, and hydrophobic aromatic scaffolds. Flavonoids and polyphenols therefore represent promising structural frameworks for the design and development of natural PDE5 inhibitors.

## 4 Conclusion

This study provides a comprehensive in silico evaluation of 76 natural compounds derived from Algerian medicinal plants as potential PDE5 inhibitors for the treatment of erectile dysfunction (ED). Drug‐likeness and toxicity predictions revealed that the majority of compounds complied with pharmacokinetic rules and exhibited low toxicity, underscoring their suitability as safe therapeutic candidates. Molecular docking identified several compounds, including ellagic acid, rosmarinic acid, catechin, and salvinorin A, with binding affinities comparable to sildenafil, the reference PDE5 inhibitor. MD simulations further validated these interactions, demonstrating that catechin, rosmarinic acid, and salvinorin A formed stable complexes with PDE5, maintaining compact protein structures and stable binding throughout the 100 ns trajectories. In contrast, ellagic acid displayed higher fluctuations, suggesting reduced conformational stability. Frontier molecular orbital analysis confirmed the electronic reactivity of catechin and rosmarinic acid, supporting their strong interaction potential with PDE5. Importantly, the integrated QSAR analysis provided quantitative confirmation of these findings, revealing that effective PDE5 inhibition is driven by an optimal balance of hydrogen‐bond donor capacity, moderate polarity, and hydrophobic aromatic scaffolds. Flavonoids and polyphenols (i.e., catechin, quercetin, apigenin, and rosmarinic acid) emerged as the most promising scaffolds, with predictive modeling reinforcing their inhibitory potential and structural suitability. Taken together, these results highlight catechin, rosmarinic acid, and related flavonoids as promising natural PDE5 inhibitors with both stability and safety advantages over synthetic alternatives. This integrated workflow, combining drug‐likeness filtering, docking, MD simulations, and QSAR modeling, provides a powerful strategy for identifying natural leads in ED management and lays the foundation for future preclinical and clinical validation.

## Author Contributions

Farouk Boudou designed the study, supervised the project, managed administration, acquired funding, and wrote and critically reviewed the manuscript. Alaeddine Berkane contributed equally by developing software, performing data curation, conducting molecular docking and virtual screening, and validating results. Amal Belakredar contributed equally to conceptualization, software development, validation, and visualization, and drafted sections of the manuscript. Ahcene Keziz contributed equally by developing methodology, performing computational modeling, and analyzing results. Huda Alsaeedi selected phytochemicals, managed databases, conducted literature review, and reviewed and edited the manuscript. Brian A. Murray evaluated the study critically, provided scientific guidance, and edited the manuscript. Mikhael Bechelany supported methodology, provided computational resources, and validated results. Ahmed Barhoum supervised the project, coordinated administration, acquired funding, and edited the manuscript.

## Conflicts of Interest

The authors declare no conflicts of interest.

## Data Availability

The data that support the findings of this study are available from the corresponding author upon reasonable request.

## References

[fsn371478-bib-0001] Abdel Bar, F. M. , N. H. Abdel Fatah , Y. Amen , A. F. Halim , and H.‐E. A. Saad . 2023. “Genus Lactuca (Asteraceae): A Comprehensive Review.” Records of Natural Products 17, no. 2: 201–231. 10.25135/rnp.350.2205-2474.

[fsn371478-bib-0003] Adamu, M. , V. Naidoo , and J. N. Eloff . 2012. “Some Southern African Plant Species Used to Treat Helminth Infections in Ethnoveterinary Medicine Have Excellent Antifungal Activities.” BMC Complementary and Alternative Medicine 12, no. 1: 1–8. 10.1186/1472-6882-12-213. PMC3528481 23134805

[fsn371478-bib-0002] Adamu, M. , V. Naidoo , and J. N. Eloff . 2014. “The Antibacterial Activity, Antioxidant Activity and Selectivity Index of Leaf Extracts of Thirteen South African Tree Species Used in Ethnoveterinary Medicine to Treat Helminth Infections.” BMC Veterinary Research 10, no. 1: 1–7. 10.1186/1746-6148-10-52. 24589020 PMC3946145

[fsn371478-bib-0004] Agarwal, A. , A. Mulgund , A. Hamada , and M. R. Chyatte . 2015. “A Unique View on Male Infertility Around the Globe.” Reproductive Biology and Endocrinology 13, no. 1: 1–9. 10.1186/s12958-015-0032-1. 25928197 PMC4424520

[fsn371478-bib-0005] Aires, A. , and R. Carvalho . 2017. “Profiling of Polyphenol Composition and Antiradical Capacity of *Erica cinerea* .” Antioxidants 6, no. 3: 72. 10.3390/antiox6030072. 28930147 PMC5618100

[fsn371478-bib-0006] Alahmar, A. T. 2019. “Role of Oxidative Stress in Male Infertility: An Updated Review.” Journal of Human Reproductive Sciences 12, no. 1: 4–18. 10.4103/jhrs.JHRS_150_18. 31007461 PMC6472207

[fsn371478-bib-0007] Al‐Tameme, H. J. , A. Saad , and N. M. Al‐Khafaji . 2019. “Study of the Effect of Aqueous Water Extracts of *Pistacia lentiscus* Bark on Some Bacteria Causing Oral Infections.” Indian Journal of Public Health Research & Development 10, no. 1: 1078–1081. 10.5958/0976-5506.2019.00204.3.

[fsn371478-bib-0008] Anjum, F. , T. Mohammad , A. A. Almalki , O. Akhtar , B. Abdullaev , and M. I. Hassan . 2021. “Phytoconstituents and Medicinal Plants for Anticancer Drug Discovery: Computational Identification of Potent Inhibitors of PIM1 Kinase.” OMICS: A Journal of Integrative Biology 25, no. 9: 580–590. 10.1089/omi.2021.010. 34448628

[fsn371478-bib-0009] Babiaka, S. B. , P. A. Onguéné , B. D. Bekono , and F. Ntie‐Kangl . 2023. “In Silico and Computational Analysis of Plant Secondary Metabolites From African Medicinal Plants.” In Applications in Plant Biotechnology, 74–90. CRC Press.

[fsn371478-bib-0011] Bhalla, M. , R. Mittal , M. Kumar , and A. S. Kushwah . 2022. “Pharmacological Aspects of a Bioactive Compound Arbutin: A Comprehensive Review.” Biointerface Research in Applied Chemistry 13: 119. 10.33263/BRIAC132.119.

[fsn371478-bib-0012] Boadi, N. O. , M. Badu , N. K. Kortei , et al. 2021. “Nutritional Composition and Antioxidant Properties of Three Varieties of Carrot (*Daucus carota*).” Scientific African 12: e00801. 10.1016/j.sciaf.2021.e00801.

[fsn371478-bib-0013] Boeing, T. , K. G. Tafarelo Moreno , A. Gasparotto Junior , L. da Mota Silva , and P. de Souza . 2021. “Phytochemistry and Pharmacology of the Genus Equisetum (Equisetaceae): A Narrative Review of the Species With Therapeutic Potential for Kidney Diseases.” Evidence‐Based Complementary and Alternative Medicine 2021, no. 1: 6658434. 10.1155/2021/6658434. 33747109 PMC7954623

[fsn371478-bib-0014] Borah, B. , and J. Saikia . 2024. “Spectroscopic and Density Functional Theory Approach to Study the Interaction of Ibuprofen With Water Molecule.” Journal of Scientific Research 16, no. 3: 647–661. 10.3329/jsr.v16i3.70331.

[fsn371478-bib-0015] Cetin, A. 2021. “In Silico Studies on Stilbenolignan Analogues as SARS‐CoV‐2 Mpro Inhibitors.” Chemical Physics Letters 771: 138563. 10.1016/j.cplett.2021.138563. 33776065 PMC7983322

[fsn371478-bib-0017] Cetin, A. 2022. “Some Flavolignans as Potent Sars‐Cov‐2 Inhibitors via Molecular Docking, Molecular Dynamic Simulations and ADME Analysis.” Current Computer‐Aided Drug Design 18, no. 5: 337–346. 10.2174/1573409918666220816113516. 35975852

[fsn371478-bib-0016] Cetin, A. , E. Oguz , E. A. Kazancioglu , B. Güven , M. Z. Kazancioglu , and F. Türkan . 2025. “Synthesis of 4‐Diazocyclohexane‐Based Sulfonamide Drug Scaffolds: Investigating Enzyme Inhibition, Antioxidant, and ADMET Properties.” Journal of Molecular Structure 1332: 141708. 10.1016/j.molstruc.2025.141708.

[fsn371478-bib-0018] Chhillar, H. , P. Chopra , and M. A. Ashfaq . 2021. “Lignans From Linseed ( *Linum usitatissimum* L.) and Its Allied Species: Retrospect, Introspect and Prospect.” Critical Reviews in Food Science and Nutrition 61, no. 16: 2719–2741. 10.1080/10408398.2020.1784840. 32619358

[fsn371478-bib-0020] De Cicco, P. , G. Ercolano , C. Sirignano , et al. 2023. “Chamomile Essential Oils Exert Anti‐Inflammatory Effects Involving Human and Murine Macrophages: Evidence to Support a Therapeutic Action.” Journal of Ethnopharmacology 311: 116391. 10.1016/j.jep.2023.116391. 36948263

[fsn371478-bib-0022] Dehelean, C. A. , I. Marcovici , C. Soica , et al. 2021. “Plant‐Derived Anticancer Compounds as New Perspectives in Drug Discovery and Alternative Therapy.” Molecules 26, no. 4: 1109. 10.3390/molecules26041109. 33669817 PMC7922180

[fsn371478-bib-0023] Djerrou, Z. , H. Benyezzar‐Kenana , Z. Maameri , and L. Benhamza . 2022. “An Ethnopharmacological Survey of Medicinal Plants Used in the Traditional Treatment of Human Infertility in Eastern Algeria.” Asian Pacific Journal of Reproduction 11, no. 2: 77. 10.4103/2305-0500.341114.

[fsn371478-bib-0024] Djordjevic, O. M. , M. R. Jakovljevic , A. Markovic , et al. 2018. “Polyphenolic Contents of Teucrium Polium L. and Teucrium Scordium L. Associated With Their Protective Effects Against MMC‐Induced Chromosomal Damage in Cultured Human Peripheral Blood Lymphocytes.” Turkish Journal of Biology 42, no. 2: 152–162. 10.3906/biy-1707-36. 30814877 PMC6353277

[fsn371478-bib-0025] Đurović, S. , I. Kojić , D. Radić , et al. 2024. “Chemical Constituents of Stinging Nettle (*Urtica dioica* L.): A Comprehensive Review on Phenolic and Polyphenolic Compounds and Their Bioactivity.” International Journal of Molecular Sciences 25, no. 6: 3430. 10.3390/ijms25063430. 38542403 PMC10970493

[fsn371478-bib-0026] Dushkin, M. , M. Khrapova , G. Kovshik , et al. 2014. “Effects of Rhaponticum Carthamoides Versus Glycyrrhiza Glabra and *Punica granatum* Extracts on Metabolic Syndrome Signs in Rats.” BMC Complementary and Alternative Medicine 14: 1–9. 10.1186/1472-6882-14-33. 24444255 PMC3905158

[fsn371478-bib-0027] Ejaz, A. , S. Waliat , M. S. Arshad , et al. 2023. “A Comprehensive Review of Summer Savory (*Satureja hortensis* L.): Promising Ingredient for Production of Functional Foods.” Frontiers in Pharmacology 14: 1198970. 10.3389/fphar.2023.1198970. 37554989 PMC10406440

[fsn371478-bib-0029] Fekih, N. , H. Allali , S. Merghache , et al. 2014. “Chemical Composition and Antibacterial Activity of *Pinus halepensis* Miller Growing in West Northern of Algeria.” Asian Pacific Journal of Tropical Disease 4, no. 2: 97–103. 10.1016/S2222-1808(14)60323-6.

[fsn371478-bib-0030] Foresta, C. , N. Caretta , D. Zuccarello , et al. 2008. “Expression of the PDE5 Enzyme on Human Retinal Tissue: New Aspects of PDE5 Inhibitors Ocular Side Effects.” Eye 22, no. 1: 144–149. 10.1038/sj.eye.6702908. 17585311

[fsn371478-bib-0031] Fossatelli, L. , Z. Maroccia , C. Fiorentini , and M. Bonucci . 2023. “Resources for Human Health From the Plant Kingdom: The Potential Role of the Flavonoid Apigenin in Cancer Counteraction.” International Journal of Molecular Sciences 25, no. 1: 251. 10.3390/ijms25010251. 38203418 PMC10778966

[fsn371478-bib-0033] Ghose, A. K. , V. N. Viswanadhan , and J. J. Wendoloski . 1999. “A Knowledge‐Based Approach in Designing Combinatorial or Medicinal Chemistry Libraries for Drug Discovery. 1. A Qualitative and Quantitative Characterization of Known Drug Databases.” Journal of Combinatorial Chemistry 1, no. 1: 55–68. 10.1021/cc9800071. 10746014

[fsn371478-bib-0034] Giraldo‐Silva, L. , B. Ferreira , E. Rosa , and A. C. Dias . 2023. “ *Opuntia Ficus‐Indica* Fruit: A Systematic Review of Its Phytochemicals and Pharmacological Activities.” Plants 12, no. 3: 543. 10.3390/plants12030543. 36771630 PMC9919935

[fsn371478-bib-0035] Guay, A. T. , J. B. Perez , J. Jacobson , and R. A. Newton . 2001. “Efficacy and Safety of Sildenafil Citrate for Treatment of Erectile Dysfunction in a Population With Associated Organic Risk Factors.” Journal of Andrology 22, no. 5: 793–797. 10.1002/j.1939-4640.2001.tb02582.x. 11545291

[fsn371478-bib-0036] Hajji, N. , S. Bayar , N. Zouari , H. Altayb , H. Sebai , and K. Chaieb . 2022. “Molecular Docking Studies, Chemical Composition, Antioxidant, Cytotoxicity, Antibacterial and Antifungal Activities of Globularia Alypum Extract.” Current Bioactive Compounds 18, no. 3: 51–64. 10.2174/1573407217666210831160746.

[fsn371478-bib-0037] Ikhou, D. , F. Boudou , H. Ziani , and D. Villemein . 2024. “Synthesis, Characterization, and Antibacterial Activity of a Novel Hybrid Material Layered Double Hydroxide Doped by Diaminododecylphosphonic Acid.” Bangladesh Journal of Pharmacology 19, no. 4: 135–146. 10.3329/bjp.v19i4.79648.

[fsn371478-bib-0039] Jaradat, N. , and A. N. Zaid . 2019. “Herbal Remedies Used for the Treatment of Infertility in Males and Females by Traditional Healers in the Rural Areas of the West Bank/Palestine.” BMC Complementary and Alternative Medicine 19, no. 1: 1–12. 10.1186/s12906-019-2617-2. 31366346 PMC6668085

[fsn371478-bib-0040] Karik, U. , I. Demirbolat , Ö. Toluk , and M. Kartal . 2021. “Comparative Study on Yields, Chemical Compositions, Antioxidant and Antimicrobial Activities of Cumin (*Cuminum cyminum* L.) Seed Essential Oils From Different Geographic Origins.” Journal of Essential Oil Bearing Plants 24, no. 4: 724–735. 10.1080/0972060X.2021.1983472.

[fsn371478-bib-0042] Kemal, M. E. , B. Bakchiche , M. Kemal , et al. 2023. “Six Algerian Plants: Phenolic Profile, Antioxidant, Antimicrobial Activities Associated With Different Simulated Gastrointestinal Digestion Phases and Antiproliferative Properties.” Journal of Herbal Medicine 38: 100636. 10.1016/j.hermed.2023.100636.

[fsn371478-bib-0044] Kuzma, M. , T. Past , G. Mózsik , and P. Perjési . 2014. “Pharmacobotanical Analysis and Regulatory Qualification of Capsicum Fruits and Capsicum Extracts—A Survey.” In Capsaicin‐Sensitive Neural Afferentation and the Gastrointestinal Tract: From Bench to Bedside, edited by G. Mózsik , O. M. E. Abdel‐Salam , and K. Takeuchi , 21–74. IntechOpen. 10.5772/58812.

[fsn371478-bib-0046] Li, Q. , T. Cheng , Y. Wang , and S. H. Bryant . 2010. “PubChem as a Public Resource for Drug Discovery.” Drug Discovery Today 15: 1052–1057. 10.1016/j.drudis.2010.10.003. 20970519 PMC3010383

[fsn371478-bib-0047] Lipinski, C. A. , F. Lombardo , B. W. Dominy , and P. J. Feeney . 1997. “Experimental and Computational Approaches to Estimate Solubility and Permeability in Drug Discovery and Development Settings.” Advanced Drug Delivery Reviews 23: 3–25. 10.1016/S0169-409X(96)00423-1. 11259830

[fsn371478-bib-0049] Manoharan, N. , D. Jayamurali , R. Parasuraman , and S. N. Govindarajulu . 2021. “Phytochemical Composition, Therapeutical and Pharmacological Potential of *Nigella sativa*: A Review.” Traditional Medicine Research 6, no. 4: 32. 10.12032/TMR20210118216.

[fsn371478-bib-0050] Mansour, I. , S. Rahmani , K. Kanoun , N. Harir , and O. Kharoubi . 2021. “Chemical Composition; Antioxidant and Antibacterial Activity of *Lavandula officinalis* Flowers Essential Oil.” Egyptian Academic Journal of Biological Sciences, C Physiology & Molecular Biology 13, no. 1: 71–82. 10.21608/EAJBSC.2021.157949.

[fsn371478-bib-0051] Medda, S. , and M. Mulas . 2021. “Fruit Quality Characters of Myrtle (*Myrtus communis* L.) Selections: Review of a Domestication Process.” Sustainability 13, no. 16: 8785. 10.3390/su13168785.

[fsn371478-bib-0052] Mehra, N. , G. Tamta , and V. Nand . 2021. “A Review on Nutritional Value, Phytochemical and Pharmacological Attributes of *Foeniculum vulgare* Mill.” Journal of Pharmacognosy and Phytochemistry 10, no. 2: 1255–1263. 10.22271/phyto.2021.v10.i2q.13983.

[fsn371478-bib-0055] Mohammed, M. J. , U. Anand , A. B. Altemimi , V. Tripathi , Y. Guo , and A. Pratap‐Singh . 2021. “Phenolic Composition, Antioxidant Capacity and Antibacterial Activity of White Wormwood (*Artemisia Herba‐Alba*).” Plants 10, no. 1: 164. 10.3390/plants10010164. 33467047 PMC7830657

[fsn371478-bib-0056] Mouissi, S. , S. Bouchelaghem , and N. Djabali . 2022. “Phytochemical Study of Two Medicinal Plants (Rosmarinus Officinalis and Anthémis Nobili) From the Haddada Region (El Tarf‐Algeria).” Ukrainian Journal of Ecology 12, no. 7: 7–14. 10.15421/2022-389.

[fsn371478-bib-0057] Mustapha, A. , A. N. AlSharksi , U. A. Eze , et al. 2024. “Phytochemical Composition, In Silico Molecular Docking Analysis and Antibacterial Activity of *Lawsonia inermis* Linn Leaves Extracts Against Extended Spectrum Beta‐Lactamases‐Producing Strains of *Klebsiella pneumoniae* .” BioMed 4, no. 3: 277–292. 10.3390/biomed4030022.

[fsn371478-bib-0059] Neculai, A.‐M. , G. Stanciu , and M. Mititelu . 2023. “Determination of Active Ingredients, Mineral Composition and Antioxidant Properties of Hydroalcoholic Macerates of *Vinca minor* L. Plant From the Dobrogea Area.” Molecules 28, no. 15: 5667. 10.3390/molecules28155667. 37570636 PMC10419528

[fsn371478-bib-0060] Ness, S. , R. Martin , A. M. Kindler , et al. 2000. “Structure‐Based Design Guides the Improved Efficacy of Deacylation Transition State Analogue Inhibitors of TEM‐1 β‐Lactamase.” Biochemistry 39, no. 18: 5312–5321. 10.1021/bi992505b. 10820001

[fsn371478-bib-0062] Oselusi, S. O. , S. A. Egieyeh , and A. Christoffels . 2021. “Cheminformatic Profiling and Hit Prioritization of Natural Products With Activities Against Methicillin‐Resistant *Staphylococcus aureus* (MRSA).” Molecules 26, no. 12: 3674. 10.3390/molecules26123674. 34208597 PMC8246317

[fsn371478-bib-0063] Oyaluna, Z. E. , A. O. Abolaji , O. Bodede , et al. 2024. “Chemical Analysis of Alliin‐Rich *Allium sativum* (Garlic) Extract and Its Safety Evaluation in *Drosophila melanogaster* .” Toxicology Reports 13: 101760. 10.1016/j.toxrep.2024.101760. 39484636 PMC11525231

[fsn371478-bib-0064] Painuli, S. , C. Quispe , J. Herrera‐Bravo , et al. 2022. “Nutraceutical Profiling, Bioactive Composition, and Biological Applications of *Lepidium sativum* L.” Oxidative Medicine and Cellular Longevity 2022, no. 1: 2910411. 10.1155/2022/2910411. 35096265 PMC8791756

[fsn371478-bib-0065] Palanichamy, C. , P. Pavadai , T. Panneerselvam , et al. 2022. “Aphrodisiac Performance of Bioactive Compounds From *Mimosa pudica* Linn.: In Silico Molecular Docking and Dynamics Simulation Approach.” Molecules 27, no. 12: 3799. 10.3390/molecules27123799. 35744923 PMC9229059

[fsn371478-bib-0066] Pinheiro, G. A. , J. Mucelini , M. D. Soares , R. C. Prati , J. L. Da Silva , and M. G. Quiles . 2020. “Machine Learning Prediction of Nine Molecular Properties Based on the SMILES Representation of the QM9 Quantum‐Chemistry Dataset.” Journal of Physical Chemistry A 124, no. 47: 9854–9866. 10.1021/acs.jpca.0c06882. 33174750

[fsn371478-bib-0067] Preethi, L. , N. Ganamurali , D. Dhanasekaran , and S. Sabarathinam . 2021. “Therapeutic Use of Guggulsterone in COVID‐19 Induced Obesity (COVIBESITY) and Significant Role in Immunomodulatory Effect.” Obesity Medicine 24: 100346. 10.1016/j.obmed.2021.100346. 33942025 PMC8081575

[fsn371478-bib-0068] Puvača, N. , V. Tufarelli , and I. Giannenas . 2022. “Essential Oils in Broiler Chicken Production, Immunity and Meat Quality: Review of *Thymus vulgaris*, Origanum Vulgare, and *Rosmarinus officinalis* .” Agriculture 12, no. 6: 874. 10.3390/agriculture12060874.

[fsn371478-bib-0069] Rafieian, F. , R. Amani , A. Rezaei , A. C. Karaça , and S. M. Jafari . 2024. “Exploring Fennel (*Foeniculum vulgare*): Composition, Functional Properties, Potential Health Benefits, and Safety.” Critical Reviews in Food Science and Nutrition 64, no. 20: 6924–6941. 10.1080/10408398.2023.2176817. 36803269

[fsn371478-bib-0070] Rahman, S. U. , S. Khan , U. Sayed , H. Ali , K. Ullah , and H. Khan . 2024. “Importance of Traditional Knowledge in Modern Drug Discovery.” In Traditional Resources and Tools for Modern Drug Discovery: Ethnomedicine and Pharmacology, 77–89. Springer Nature Singapore. 10.1007/978-981-97-4600-2_4.

[fsn371478-bib-0072] Sánchez‐Gutiérrez, M. , R. Gómez‐García , E. Carrasco , I. Bascón‐Villegas , A. Rodríguez , and M. Pintado . 2022. “ *Quercus Ilex* Leaf as a Functional Ingredient: Polyphenolic Profile and Antioxidant Activity Throughout Simulated Gastrointestinal Digestion and Antimicrobial Activity.” Journal of Functional Foods 91: 105025. 10.1016/j.jff.2022.105025.

[fsn371478-bib-0073] Sang, S. , Z. Yang , L. Wang , X. Liu , H. Lin , and J. Wang . 2018. “SemaTyP: A Knowledge Graph Based Literature Mining Method for Drug Discovery.” BMC Bioinformatics 19, no. 1: 1–11. 10.1186/s12859-018-2167-5. 29843590 PMC5975655

[fsn371478-bib-0074] Shamloul, R. , and H. Ghanem . 2013. “Erectile dysfunction.” Lancet 381, no. 9861: 153–165. 10.1016/S0140-6736(12)60520-0. 23040455

[fsn371478-bib-0076] Skanes, B. , K. Warriner , and R. S. Prosser . 2021. “Hazard Assessment Using an In‐Silico Toxicity Assessment of the Transformation Products of Boscalid, Pyraclostrobin, Fenbuconazole and Glyphosate Generated by Exposure to an Advanced Oxidative Process.” Toxicology In Vitro 70: 105049. 10.1016/j.tiv.2020.105049. 33171224

[fsn371478-bib-0077] Sumera , F. Anwer , M. Waseem , et al. 2022. “Molecular Docking and Molecular Dynamics Studies Reveal Secretory Proteins as Novel Targets of Temozolomide in Glioblastoma Multiforme.” Molecules 27: 7198. 10.3390/molecules27217198. 36364024 PMC9653723

[fsn371478-bib-0078] Tahraoui, A. , J. El‐Hilaly , Z. Israili , and B. Lyoussi . 2007. “Ethnopharmacological Survey of Plants Used in the Traditional Treatment of Hypertension and Diabetes in South‐Eastern Morocco (Errachidia Province).” Journal of Ethnopharmacology 110, no. 1: 105–117. 10.1016/j.jep.2006.09.011. 17052873

[fsn371478-bib-0079] Thomford, N. E. , K. Dzobo , D. Chopera , et al. 2015. “Pharmacogenomics Implications of Using Herbal Medicinal Plants on African Populations in Health Transition.” Pharmaceuticals 8, no. 3: 637–663. 10.3390/ph8030637. 26402689 PMC4588186

[fsn371478-bib-0081] Ukiya, M. , T. Akihisa , K. Yasukawa , et al. 2002. “Anti‐Inflammatory and Anti‐Tumor‐Promoting Effects of Cucurbitane Glycosides From the Roots of *Bryonia dioica* .” Journal of Natural Products 65, no. 2: 179–183. 10.1021/np010423u. 11858752

[fsn371478-bib-0083] Vella, F. M. , D. Pignone , and B. Laratta . 2024. “The Mediterranean Species Calendula Officinalis and *Foeniculum vulgare* as Valuable Source of Bioactive Compounds.” Molecules 29, no. 15: 3594. 10.3390/molecules29153594. 39124999 PMC11314138

[fsn371478-bib-0084] Verma, A. K. , S. F. Ahmed , M. S. Hossain , et al. 2021. “Molecular Docking and Simulation Studies of Flavonoid Compounds Against PBP‐2a of Methicillin‐Resistant *Staphylococcus aureus* .” Journal of Biomolecular Structure and Dynamics 40: 1–17. 10.1080/07391102.2021.1944911. 34243699

[fsn371478-bib-0085] Vinciguerra, V. , F. Rojas , V. Tedesco , G. Giusiano , and L. Angiolella . 2019. “Chemical Characterization and Antifungal Activity of *Origanum vulgare* , *Thymus vulgaris* Essential Oils and Carvacrol Against Malassezia Furfur.” Natural Product Research 33, no. 22: 3273–3277. 10.1080/14786419.2018.1468325. 29726703

[fsn371478-bib-0087] Wu, M. , M. Liu , F. Wang , et al. 2022. “The Inhibition Mechanism of Polyphenols From *Phyllanthus emblica* Linn. Fruit on Acetylcholinesterase: A Interaction, Kinetic, Spectroscopic, and Molecular Simulation Study.” Food Research International 158: 111497. 10.1016/j.foodres.2022.111497. 35840206

[fsn371478-bib-0089] Yang, Z. , Z. Guo , J. Yan , and J. Xie . 2024. “Nutritional Components, Phytochemical Compositions, Biological Properties, and Potential Food Applications of Ginger (*Zingiber officinale*): A Comprehensive Review.” Journal of Food Composition and Analysis 128: 106057. 10.1016/j.jfca.2024.106057.

[fsn371478-bib-0090] Yuan, S. , H. S. Chan , and Z. Hu . 2017. “Using PyMOL as a Platform for Computational Drug Design.” Wiley Interdisciplinary Reviews: Computational Molecular Science 7, no. 2: e1298. 10.1002/wcms.1298.

[fsn371478-bib-0091] Zahedifar, M. , and S. Najafian . 2023. “Variation of Antioxidant Activity and Phenolic Compositions of *Marrubium vulgare* L. as Influenced by Organic Acids.” Journal of Medicinal Plants 22, no. 87: 77–88. 10.61186/jmp.22.87.77.

[fsn371478-bib-0092] Zam, W. , C. Quispe , J. Sharifi‐Rad , et al. 2022. “An Updated Review on the Properties of *Melissa officinalis* L.: Not Exclusively Anti‐Anxiety.” Frontiers in Bioscience 14, no. 2: 16. 10.31083/j.fbs1402016. 35730441

[fsn371478-bib-0094] Zhou, T.‐Q. , Z.‐Z. Wei , J.‐R. Zhang , et al. 2023. “Phytochemical Constituents From the Seeds of Capsella Bursa‐Pastoris and Their Antioxidant Activities.” Plant Foods for Human Nutrition 78, no. 4: 776–782. 10.1007/s11130-023-01097-z. 37668768

[fsn371478-bib-0096] Zouaoui, Z. , A. Ennoury , N. Nhhala , et al. 2024. “Juniperus Oxycedrus L. Phytochemistry and Pharmacological Properties: A Review.” Scientific African: e02361. 10.1016/j.sciaf.2024.e02361.

